# Combined Metabolomic Analysis of Plasma and Urine Reveals AHBA, Tryptophan and Serotonin Metabolism as Potential Risk Factors in Gestational Diabetes Mellitus (GDM)

**DOI:** 10.3389/fmolb.2017.00084

**Published:** 2017-12-21

**Authors:** Miriam Leitner, Lena Fragner, Sarah Danner, Nastassja Holeschofsky, Karoline Leitner, Sonja Tischler, Hannes Doerfler, Gert Bachmann, Xiaoliang Sun, Walter Jaeger, Alexandra Kautzky-Willer, Wolfram Weckwerth

**Affiliations:** ^1^Gender Medicine Unit, Division of Endocrinology and Metabolism, Department of Internal Medicine III, Medical University of Vienna, Vienna, Austria; ^2^Department of Ecogenomics and Systems Biology, University of Vienna, Vienna, Austria; ^3^Vienna Metabolomics Center, University of Vienna, Vienna, Austria; ^4^Department of Clinical Pharmacy and Diagnostics, University of Vienna, Vienna, Austria

**Keywords:** diabetes, serotonin, GC-MS, SID-MS, urine, plasma, tryptophan metabolism, nutrition

## Abstract

Gestational diabetes mellitus during pregnancy has severe implications for the health of the mother and the fetus. Therefore, early prediction and an understanding of the physiology are an important part of prenatal care. Metabolite profiling is a long established method for the analysis and prediction of metabolic diseases. Here, we applied untargeted and targeted metabolomic protocols to analyze plasma and urine samples of pregnant women with and without GDM. Univariate and multivariate statistical analyses of metabolomic profiles revealed markers such as 2-hydroxybutanoic acid (AHBA), 3-hydroxybutanoic acid (BHBA), amino acids valine and alanine, the glucose-alanine-cycle, but also plant-derived compounds like sitosterin as different between control and GDM patients. PLS-DA and VIP analysis revealed tryptophan as a strong variable separating control and GDM. As tryptophan is biotransformed to serotonin we hypothesized whether serotonin metabolism might also be altered in GDM. To test this hypothesis we applied a method for the analysis of serotonin, metabolic intermediates and dopamine in urine by stable isotope dilution direct infusion electrospray ionization mass spectrometry (SID-MS). Indeed, serotonin and related metabolites differ significantly between control and GDM patients confirming the involvement of serotonin metabolism in GDM. Clustered correlation coefficient visualization of metabolite correlation networks revealed the different metabolic signatures between control and GDM patients. Eventually, the combination of selected blood plasma and urine sample metabolites improved the AUC prediction accuracy to 0.99. The detected GDM candidate biomarkers and the related systemic metabolic signatures are discussed in their pathophysiological context. Further studies with larger cohorts are necessary to underpin these observations.

## Introduction

Gestational diabetes mellitus (GDM) is defined as glucose intolerance with onset or new recognition in pregnancy (Kautzky-Willer et al., 2004, 2016b). The prevalence of GDM is rising worldwide, reaching up to 25.1% (Zhu and Zhang, 2016). Therefore, GDM is the most common form of metabolic complication in pregnancy (Erem et al., 2015) and is associated with fetal macrosomia (Witkop et al., 2009), hyperbilirubinemia, and in consequence shoulder dystocia (Xiong et al., 2001; HAPO Study Cooperative Research Group, 2008; Yogev et al., 2009) as well as maternal morbidity (hypertension, polyhydramnion, infection). Women who had GDM have an elevated risk to develop Diabetes mellitus Type 2 (T2DM) or cardiovascular disease (Tobias et al., 2011), as well as hyperlipidemia or obesity (Bartha et al., 2008; Clausen et al., 2009; Landon et al., 2009; Gillman et al., 2010; Harreiter et al., 2014) in later life, whereas their children have a higher risk for obesity or impaired fasting glucose (Silverman et al., 1995). Further, GDM affects the psychological health of the mother and the child: GDM increases a woman's risk of postpartum depression 4-fold (Hinkle et al., 2016), and postpartum depression is decreased with treatment of GDM (Crowther et al., 2005; Beucher et al., 2010). Intrauterine exposure to hyperglycemia is linked to an increased risk for neuropsychiatric and neurodevelopmental disorders of the offspring (Xiang et al., 2015; Nahum Sacks et al., 2016).

Early detection of GDM and treatment can reduce the risk for mother and child. The current gold standard of diagnosing GDM is an oral glucose tolerance test (oGTT) between 24 and 28 weeks of gestation. However, detection of women at risk even earlier during pregnancy would be important to enable early lifestyle modification or even drug treatment in order to improve perinatal outcomes of these women. In addition other markers than glucose could be useful in identification of women and neonates at greatest risk. Therefore, the identification of new reliable and easily accessible biomarkers for earlier diagnosis of women with metabolic alterations during pregnancy would be of great value.

When the fetus starts to grow the maternal metabolism changes. More and more energy is required to ensure the growth of the unborn child. For this reason the maternal metabolic state must be modified in different and multiple ways to afford the energy demand. Among the altered mechanisms, an adaptation of hormones appears, such as insulin, serotonin (5-hydroxytryptamin, 5HT), hepatocyte growth factor (HGF) and cortisol (Ernst et al., 2011). Moreover transcription factors and cell cycle regulators cause a change in the metabolism of the mother during pregnancy (Ernst et al., 2011). These modulations are essential physiological elements for the normal progress of pregnancy, but can implicate pathological diabetic condition if they are disrupted. Although the altered mechanisms in GDM and overt diabetes are similar and pathways can be deduced, the conditions and the pathophysiology differ, and more research is needed on the most common but heterogeneous form, namely GDM (International Association of Diabetes in Pregnancy Study Group Working Group on Outcome et al., 2015; Kautzky-Willer et al., 2016a; Simmons et al., 2016; Rosta et al., 2017).

Metabolomics has the capacity to detect early deregulations and disruptions in metabolism associated with diseases or disorders. To investigate physiological processes and to develop (early) diagnostics, metabolomics is one of the most promising technologies (Bain et al., 2009; Pinto et al., 2015; Allalou et al., 2016). In case of glucose disrupted states (like GDM), there are novel findings regarding hitherto inconspicious hormones like melatonin or serotonin which arouse interest (Ernst et al., 2011) and legitimate closer examinations of the metabolome and hormone levels in GDM. Therefore we used untargeted and targeted metabolomic technology for the combined investigation of the metabolome in 32 pregnant women with and without GDM in blood plasma and urine and performed a metabolite profiling. Differences in the profiles between the case and control group were detected pointing to potential biomarkers and physiological processes for early GDM. Complementary to these metabolite markers the body mass index (BMI) and week of pregnancy (see Supplementary Table S1) were monitored. Combined regression analysis of the latter, blood plasma and urine metabolites improved the AUC (Area under curve) prediction accuracy to 0.99. In this paper we discuss corresponding hypotheses and assumptions for detected alterations in metabolic pathways for a better understanding of the metabolomic changes occuring in GDM.

## Materials and methods

### Study population

Participants were recruited from the outpatient clinic at the Medical University of Vienna. We investigated 14 women with GDM and 18 women without GDM (nGDM). The participants were 21–41 years old and in 12^th^−26^th^ week of pregnancy (see Supplementary Table S1 also for BMI). The study was performed in accordance with the ethical principles of the Declaration of Helsinki II and was approved by the local ethics committee (Ethics Committee of the Medical University of Vienna). All participants gave their written informed consent.

### GDM definition, BMI and sample preparation

GDM was diagnosed with a standard 2 h 75 g oral glucose tolerance test (oGTT), according to the International Association of Diabetes and Pregnancy Study Groups (IADPSG) criteria (International Association of Pregnancy Study Groups Consensus Panel et al., 2010; Colagiuri et al., 2014). Eighteen control and fourteen GDM cases were defined. Urine samples were taken before the oGTT and urine was stored at −20°C. Blood samples were taken at three time points of the oGTT (0, 60, and 120 min after glucose intake). Plasma samples were prepared by centrifugation. Coagulation was avoided with an anticoagulant, ethylenediaminetetraacetic acid (EDTA). Samples were stored at −20°C until measurement. BMI was calculated as weight (kg) divided by the square of height (m^2^).

### Plasma sample extraction, derivatization, GC-MS analysis, identification and quantification

Extraction and derivatization were performed slightly modified according to Weckwerth et al. (2004b). Chemicals were purchased from Sigma-Aldrich (Austria) if not stated otherwise: Methanol (CHROMASOLV®, HPLC grade), chloroform (anhydrous ≥99%, stabilized with 0.5–1% ethanol). Water was double-distilled and deionized (Milli-Q water® Advantage A10, Austria).

Samples were extracted in batches of 12, each including a pooled quality control sample and a blank processed in the same way. The pooled sample was a mixture of randomly chosen blood aliquots of half of the women, containing most of the metabolites expected in plasma (Dunn et al., 2011). Extraction was done by adding 200 μl of plasma to 1.7 ml of pre-chilled (−20°C) extraction mixture: methanol/chloroform/water (2.5:1:0.5 (v/v/v)) (MCW) and internal standard was spiked (1 μmol of D-Sorbitol-^13^C_6_, 98 atom % ^13^C, Campro Scientific, Germany). Sample tubes were agitated for 10 s and incubated for 8 min on ice. Samples were centrifuged for 4 min at 14,000 g at 4°C. The one-phase-supernatant was collected in a new Eppendorf tube, shortly vortexed and divided into two equal aliquots, corresponding to 100 μl plasma each. Samples were dried using a speed vac (Savant^TM^, Thermo Scientific, Austria) and stored at −80°C until measurements. One aliquot of each sample was used for further GC-MS analyses and metabolites were derivatized before measurement in two steps. Dried extracts were acclimated at room temperature for 10 min, dissolved in 20 μl solution of 40 mg/ml methoxyamine hydrochloride (CH3ONH2^*^HCL) in pyridine and incubated for 90 min at 30°C on a thermo shaker (550 rpm). Subsequently, 80 μl of N-methyl-N-(trimethylsilyl) trifluoroacetamide (MSTFA) (Macherey Nagel, Germany) were added and incubated for 30 min at 37°C. After centrifugation (2 min at 14,000 g), the supernatant was transferred to glass vials with micro inserts, closed with crimp caps and measured by gas chromatography coupled to mass spectrometry (GC-MS). GC-MS analyses and data validation were performed according to previous publications (Mari et al., 2013; Prezelj et al., 2016) with slight modifications on a ThermoFisher Trace GC coupled to a Triple Quadrupole mass analyzer (Thermo Scientific TSQ Quantum GC™, Bremen, Germany). Each batch included randomly chosen samples, a blank, a pooled quality control sample and a pure non derivatized alkane standard mixture of even-numbered n-alkanes (C10-C40, each 50 mg/L in hexane) for retention index (RI) determination. One microliter of sample was injected in splitless mode at a constant injector temperature of 230°C using a deactivated stainless steel Siltek liner (Restek Corp., USA). GC separation was performed on a HP-5MS capillary column (30 m × 0.25 mm × 0.25 μm) (Agilent Technologies, CA) at a constant helium flow rate of 1 mL min^−1^. Initial oven temperature was set to 70°C and held for 1 min, followed by a ramp to 76°C at 1°C min^−1^ and a second ramp at 6°C min^−1^ to 350°C held for 1 min. Transfer line temperature was set to 340°C and post run temperature to 325°C for 10 min. The quadrupole mass analyzer was used in full scan mode with a scan range of *m/z* 40–600 Th and a scan time of 250 msec. Electron impact (EI) ionization was performed at 70 eV with 50 μA emission current and ion source temperature was set to 250°C. Metabolite derivatives were identified by matching retention time as well as mass spectra with those of reference standards and by comparison of alkane based retention indices with an in house mass spectral library, as well as the GMD library (Kopka et al., 2005). Metabolites were considered as annotated with a spectral match factor higher than 850 (NIST MS Search 2.0 Program algorithm) and RI-deviation lower than 4%. Deconvolution and RI-deviation calculation was performed with AMDIS (Stein, 1999) and quantification with LC-Quan 2.6.0 (Thermo Fisher Scientific Inc.). Peak areas of a specific ion of a compound (quant *m/z*) were normalized to the ^13^C-sorbitol peak within each run. More detailed information and a list of quantified analytes can be found in Supplementary Table S1.

### Stable isotope diluted direct infusion electrospray ionization mass spectrometry (SID-MS) analysis of serotonin metabolism in urine

If not stated otherwise solvents were purchased from Sigma-Aldrich (Austria) in high quality (CHROMASOLV®, HPLC grade), formic acid (ROTIPURAN®) was purchased from Carl Roth (Germany), acetic acid from Fisher Scientific (Austria) and water was double-distilled Milli-Q water as stated above.

Urine samples were purified prior analyses by solid phase extraction (SPE) using a slightly modified protocol according to Moriarty et al. (2011). Reaction tubes (15 ml, Greiner bio-one) were prepared by cutting a hole into the lid in the size of a SPE C18 cartridge (InertSep^TM^ 100 mg/1 ml, GL Sciences, Japan). SPE cartridges were inserted into the tubes and conditioned with 3 × 1 ml methanol, followed by 3 × 1 ml of acidified water to ph 3.5 with acetic acid. Before loading, urine samples were diluted by adding 500 μl sample to 800 μL acidified water (ph 3.5) in a 2 ml reaction tube including internal standard serotonin-*d*_4_ (1 μMol) (98 atom% D, CDN Isotopes, Canada). After vortexing and centrifugation for 5 min at 14,000 g the supernatant was loaded on the SPE cartridge and washed with 1 ml 5% (v/v) methanol solution. Metabolites were eluted with 5 × 1 ml 0.1 M ammonium acetate in methanol. SPE cartridge tubes were centrifuged after each solvent or sample loading step at 1,000 g for 30 sec at 4°C. The collected eluate was dried for 2 h to absolute dryness under a gentle stream of nitrogen (N_2_ 99.999%) and stored at −20°C.

Purified and dried urine samples were dissolved in 200 μL 0.1% formic acid in methanol, centrifuged at 21,000 g and supernatant were transferred to glass vials with micro-inserts which were crimped with pre-perforated lids. Samples were kept at 4°C during all preparation steps of extraction, purification and analyses.

Targeted SID-MS detection and quantification of serotonin-melatonin-tryptophan pathway metabolites were performed on an Orbitrap Elite mass spectrometer (Thermo Fisher Scientific, USA). Direct infusion of samples was performed using a nano UHPLC pump equipped with an autosampler (Dionex UltiMate 3000 RSLCnano UHPLC pump, Thermo Fisher Scientific) by an isocratic flow. To prevent clogging of the nano spray needle, a PicoChip^TM^ nano emitter system for Infusion (New objective Inc., USA) was used and ionization was conducted using a nano spray ionisation source (NSI). In the following, instrument descriptions and detailed parameters are given. Injection volume was 5 μl, isocratic flow was performed at a flow rate of 500 nL/min, with 60% mobile phase A: 0.1% formic acid and 40% mobile phase B (90% ACN, 10% H_2_O, +0.1% formic acid) with a total run time of 15 min. Tip size of the PicoChip^TM^ nano emitter was 15 μm and NSI source parameters were as follows: source voltage 1.9 kV, source current 100 μA, sheath gas 0, aux gas 0 and capillary temperature 275°C.

Accurate mass analysis was performed using the Orbitrap FTMS mass analyzer using the lock mass option in MS and MS/MS mode. Ions of cyclomethicone N5 (m/z = 371.101230) were used for internal mass calibration. Data dependent MS^2^ scan experiments of a target parent mass list was performed at a mass resolution of 120,000; scan event 1 was performed in full scan mode and a scan range of *m/z* 110–600 Th, MS_2_ fragmentation of triggered precursor masses in scan event 2 was performed by collision induced dissociation (CID) with 50 eV normalized collision energy. Precursor ions of the target analytes serotonin, 5-hydroxyindolic acetic acid, N-acetylserotonin, 5-methoxytryptamin, melatonin, 6-hydroxymelatonin, L-tryptophan, 5-hydroxytryptophan and dopamine, as well as observed MS^2^ fragments are summarized in Table 1. Mass calibration was performed once a week. For quantification average intensities of MS2 product ions were used and normalized to the stable isotope labeled internal standard serotonin-*d*_4_.

**Table 1:** Metabolite and mass spectral information of targeted SID-MS analysis.

**Metabolite**	**Synonym**	**Abbreviation**	MSI^*^	**PubChem CID**	**InChI**	**CAS**	**Molecular formula**	**Monoisotopic mass [g/mol]**	Adduct/in source fragment^**^	**Precursor target list m/z [Th]**	MS2 fragments^***^
Serotonin	5-Hydroxytryptamine	5-HT	1	5202	InChI = 1S/C10H12N2O/c11-4-3-7-6-12-10-2-1-8(13)5-9(7)10/h1-2,5-6,12-13H,3-4,11H2	50-67-9	C_10_H_12_N_2_O	176.095	[M+H]^+^	177.10136	131.00, 121.10, 159.12, 135.08, 107.09
									[M+H]^+^ -NH_3_	160.07474	132.08, 133.06, 115.05
5-hydroxyindoleacetic acid	5-Hydroxyindole-3-acetic acid, 5-Hydroxyindoleacetate	5-HIAA	1	1826	InChI = 1S/C10H9NO3/c12-7-1-2-9-8(4-7)6(5-11-9)3-10(13)14/h1-2,4-5,11-12H,3H2,(H,13,14)	54-16-0	C_10_H_9_NO_3_	191.058	[M+H]^+^	192.06548	146.06, 119.05, 174.15, 164.07, 110.06
									by peak	146.06009	118.07, 100.08, 113.95, 131.96, 146.06
N-acetylserotonin	N-Acetyl-5-hydroxytryptamine, Normelatonin		1	903	InChI = 1S/C12H14N2O2/c1-8(15)13-5-4-9-7-14-12-3-2-10(16)6-11(9)12/h2-3,6-7,14,16H,4-5H2,1H3,(H,13,15)	1210-83-9	C_12_H_14_N_2_O_2_	218.106	[M+H]^+^	219.11217	173.05, 191.06, 160.08,
									[M+H]^+^ -NH_3_	202.08580	160.08, 184.08, 102.09, 174.09
5-methoxytryptamine	Methoxytryptamine		1	1833	InChI = 1S/C11H14N2O/c1-14-9-2-3-11-10(6-9)8(4-5-12)7-13-11/h2-3,6-7,13H,4-5,12H2,1H3	608-07-1	C_11_H_14_N_2_O	190.111	[M+H]^+^	191.11743	145.02, 163.03, 173.13, 191.02
									[M+H]^+^ -NH_3_	174.09076	159.07, 143.07
Melatonin	N-Acetyl-5-methoxytryptamine		1	896	InChI = 1S/C13H16N2O2/c1-9(16)14-6-5-10-8-15-13-4-3-11(17-2)7-12(10)13/h3-4,7-8,15H,5-6H2,1-2H3,(H,14,16)	73-31-4	C_13_H_16_N_2_O_2_	232.121	[M+H]^+^	233.12802	174.09, 216.10
									[M+H]^+^ -NH_3_	216.10159	174.09, 198.09, 116.11
6-hydroxymelatonin	6-OH-melatonin		1	1864	InChI = 1S/C13H16N2O3/c1-8(16)14-4-3-9-7-15-11-6-12(17)13(18-2)5-10(9)11/h5-7,15,17H,3-4H2,1-2H3,(H,14,16)	2208-41-5	C_13_H_16_N_2_O_3_	248.116	[M+H]^+^	249.12321	190.09, 232.10
									[M+H]^+^ -NH_3_	232.09694	190.09, 217.07
Tryptophan	L-tryptophan	Trp	1	6305	InChI = 1S/C11H12N2O2/c12-9(11(14)15)5-7-6-13-10-4-2-1-3-8(7)10/h1-4,6,9,13H,5,12H2,(H,14,15)/t9-/m0/s1	73-22-3	C_11_H_12_N_2_O_2_	204.09	[M+H]^+^	205.09679	188.07, 187.02, 159.03, 177.13, 163.11, 135.12, 121.10, 145.10
									[M+H]^+^ -NH_3_	188.07033	146.06, 144.08
5-hydroxytryptophan	5-OH-tryptophan		1	144	InChI = 1S/C11H12N2O3/c12-9(11(15)16)3-6-5-13-10-2-1-7(14)4-8(6)10/h1-2,4-5,9,13-14H,3,12H2,(H,15,16)	56-69-9	C_11_H_12_N_2_O_3_	220.085	[M+H]^+^	221.09175	204.07, 90.98, 159.98
									[M+H]^+^ -NH_3_	204.06529	162.05, 186.05
Dopamine	Dopamine		1	681	InChI = 1S/C8H11NO2/c9-4-3-6-1-2-7(10)8(11)5-6/h1-2,5,10-11H,3-4,9H2	51-61-6	C_8_H_11_NO_2_	153.079	[M+H]^+^	154.08560	137.06, 140.03, 122.02
									[M+H]^+^ -NH_3_	137.05899	119.05, 91.05, 109.06
Serotonin-d_4_	[2H4]-Serotonin		1	71752180	InChI = 1S/C10H12N2O/c11-4-3-7-6-12-10-2-1-8(13)5-9(7)10/h1-2,5-6,12-13H,3-4,11H2/i3D2,4D2	n.a.	C_10_H_12_N_2_O	180.12	[M+H]^+^	181.12735	164.10, 162.08, 161.08
									[M+H]^+^ -NH_3_	164.10080	136.11

* *MSI level according to Sumner et al., 2007, Level I was considered when identification was confirmed with a standard measured on the same instrument with the same method on MS1 and MS2 level*.

** *For many metabolites an in source fragmentation resulting in a loss of an NH_3_ was observed*.

*** *Most abundant MS2 fragments (>10% rel. Intensity) using collision induced dissociation (CID) at collision energy = 50 eV*.

### Statistical analysis

Log transformation and data normalization using internal standards to avoid interday tuning differences were applied. Two tailed unpaired *t*-test was used to compare the differences in metabolites between the control and the GDM-group. A *p*-value of less than 0.05 was regarded to be statistically significant. Further, the data were analysed with principal component analysis (PCA) and ANOVA integrated in the statistical toolbox COVAIN (Sun and Weckwerth, 2012) to find group separations and significant metabolite changes between control and GDM. Additionally, the data matrices are analyzed with partial least squares discriminant analysis (PLS-DA) using SIMCA-PR (Umetrics, Sweden). The PLS-DA model employed 7 cross validation groups, assigning every 7th observation to the same group, and grouping similar observations in the same groups. In Supplementary Figure S1 Q2(cum) and R2(cum) are given as indicators for goodness of fit and the predictive quality of all three time points. Variable importance selection was conducted with a VIP-analysis (variable importance in the projection).

Clustered correlation coefficient matrix visualization was performed with an in house written Matlab script (Matlab R2016b, version 9.1, ® Natick, Massachusetts, United States). The script is available upon request from the corresponding author.

BMI, week of pregnancy and blood and urine sample metabolites were analyzed by LASSO regression (least absolute shrinkage and selection operator). For 21 patients (10 GDM, 11 normal) with serotonin and intermediates measurements, we built statistical models to predict GDM from a combination of metabolomics (131 metabolites) as well as serotonin data (8 compounds). First, metabolomics data were log-transformed. Second, all variables were standardized by the *z*-score transformation. Third, LASSO regression combined with a linear SVM (support vector machine) classifier, was applied to select the best subset of variables that achieve highest prediction accuracy. LASSO is a regularization regression method that penalizes the absolute size of the regression coefficients to avoid overfitting that is common in many regression problems. Here, LASSO was applied with a range of lambda values, or the regularization parameter. For each lambda value, a subset of variables are selected. Then a linear SVM model was built using these variables for the two-class (GDM = 1, nGDM = 0) classification problem. The concept of SVM is to maximize the margin that distinguish groups of data by fitting a “hyperplane.” It proves to work well with biological data where the sample size is small. The SVM model was evaluated by five-fold cross validation (CV). After trying all lambda values, the best subset of variables were selected from the model with minimal CV error. All the analysis was done by the Statistics and Machine Learning Toolbox in Matlab R2016b (version 9.1, ® Natick, Massachusetts, United States).

## Results

### Plasma metabolite analysis of control and GDM patients during an oral glucose tolerance test (oGTT)

**Figure 1:**
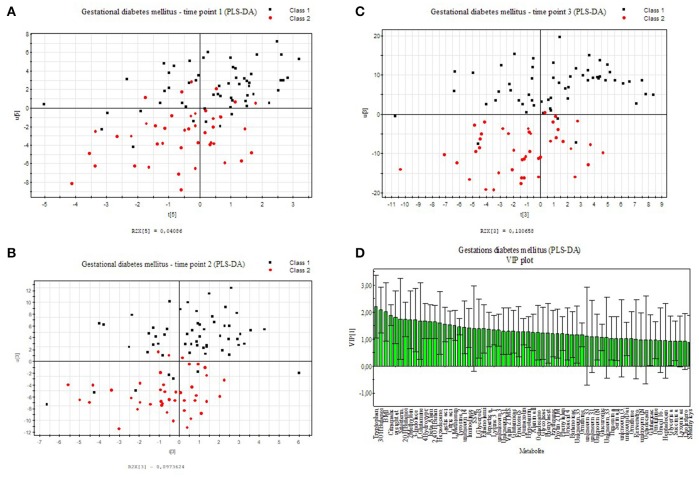
PLS-DA model built for two classes, control and GDM for three different time points of the oral glucose tolerance test (oGTT). Healthy pregnant women are labeled by black squares (class 1) and GDM individuals are labeled with red dots (class 2). **(A)** 0 h, **(B)** 1 h, **(C)** 2 h **(D)** VIP projection of the variables according to the scores plot of **B**.

We applied an universal integrative protocol for extraction and analysis of metabolites from plasma samples (Weckwerth et al., 2004b). After extraction with MCW (methanol, chloroform, water in a one phase mixture) no further phase separation was performed and the complete MCW extract was injected into GC-MS. With the modified protocol 131 annotated and putative metabolites were detected (see Supplementary Table S1). The metabolite data were further analyzed by PLS-DA (see Figure 1). Healthy pregnant women are labeled by black squares (class 1) and diabetic individuals are labeled with red dots (class 2). PLS-DA revealed a separation between case and control groups in all three time points of the oGTT (Figures 1A–C). To detect the metabolites, which are most responsible for the separation in the PLS-scatter plots a VIP analysis has been performed. The pattern of the corresponding VIP analysis (Figure 1D) illustrates the metabolites importantance projection for the sample separation seen in the PLS-DA plot in Figure 1B. VIP values larger than one indicate important metabolites, which were grouped into classes. The VIP plot (Figure 1D), sorted by importance, showed the metabolites tryptophan and 3-hydroxybutanoic acid as most discriminatory between GDM and control. Further interesting metabolite markers discriminating GDM and control are discussed below.

### Amino acids, fatty acids, organic acids, sugars, and steroids discriminate control and GDM

In Supplementary Figure S1 selected boxplots of statistically significant organic acids, branched-chain amino acids (BCAA), and further amino acids are shown. Valine, alanine and β-alanine differ significantly between patients with and without GDM. Several fatty acids, hydroxy acids and other organic acids as well as ketone bodies and sugars show differences. 2- and 3-hydroxybutanoic acid (α- and β-hydroxybutyric acid, AHBA and BHBA) differ significantly (see boxplots Supplementary Figure S1).

The intermediates of the tricarboxylic acid cycle (TCA) show significant differences between the GDM- group and the control-group. The potentially identified plant sterol β-sitosterol, derived from diet, and cholesterol were found significantly changed. β-Sitosterol had also high loadings in the VIP analysis (see Figure 1D).

### Serotonin metabolism is changed in GDM

**Figure 2:**
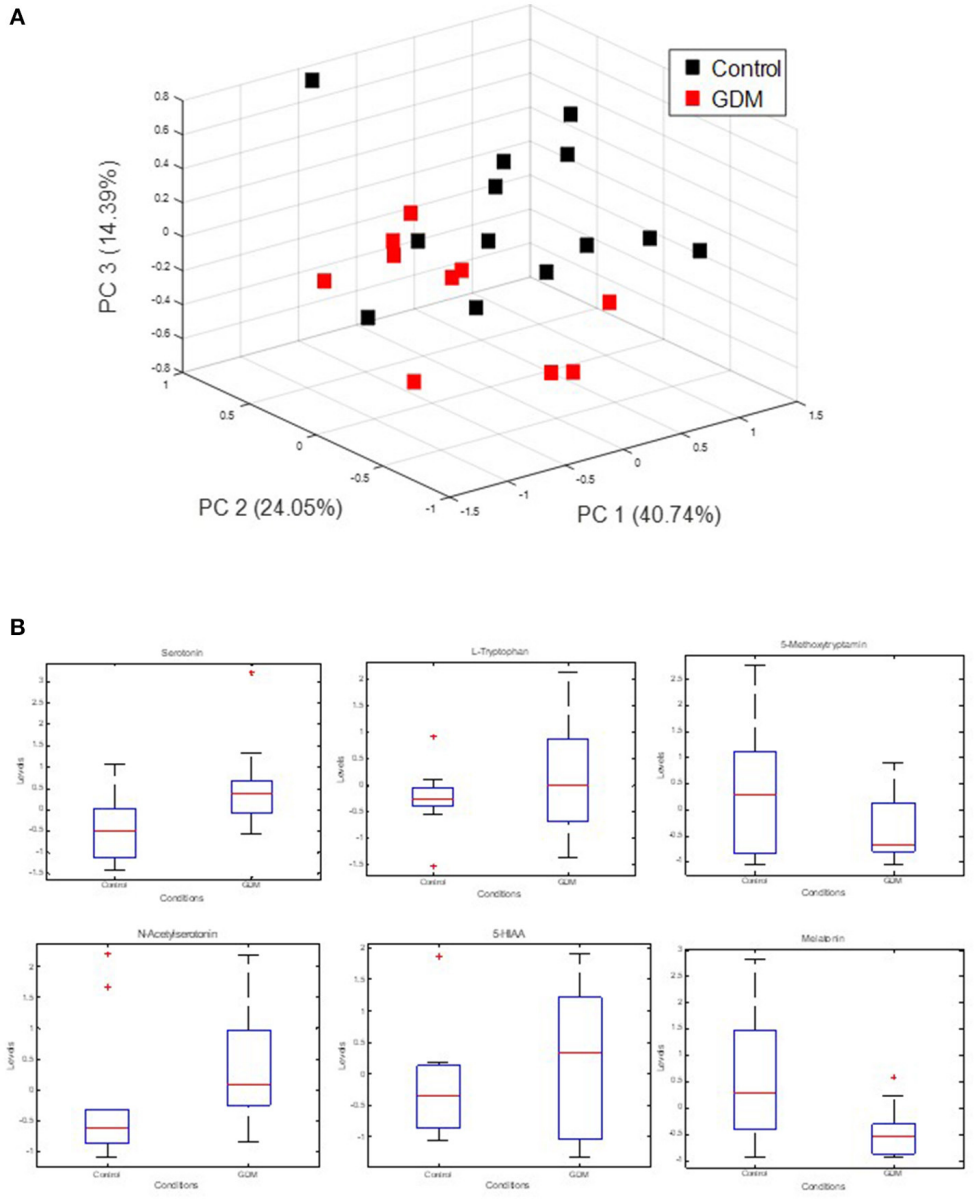
Measurement of extracellular serotonin metabolic intermediates and dopamine in urine of GDM patients. **(A)** Principal component analysis of serotonin metabolic intermediates. **(B)** Boxplots of serotonin metabolic intermediates in urine of GDM vs. control groups.

The strong VIP loadings of tryptophan pointed us to serotonin metabolism. Because serotonin and intermediates are only weakly covered by GC-MS we applied a stable isotope dilution direct-infusion method (SIDE-MS assay, see section Materials and Methods). We applied this assay to the analysis of urine samples from the GDM and the control group. Urine analysis is a non-invasive technique complementing any other profiling method with lowest costs. In Figure 2A a principal component analysis demonstrates that serotonin metabolism is altered in GDM patients in comparison to the control-group. In Figure 2B boxplots are shown of selected intermediates with high loadings.

### Integrative analysis of plasma and urine metabolomics

Integrative analysis of plasma and urine metabolite profiles was performed in two steps: (i) a systemic analysis using metabolite correlation networks of control and GDM cases, and (ii) using a linear regression method to determine the best set of candidate biomarker for accurate prediction of GDM cases.

**Figure 3:**
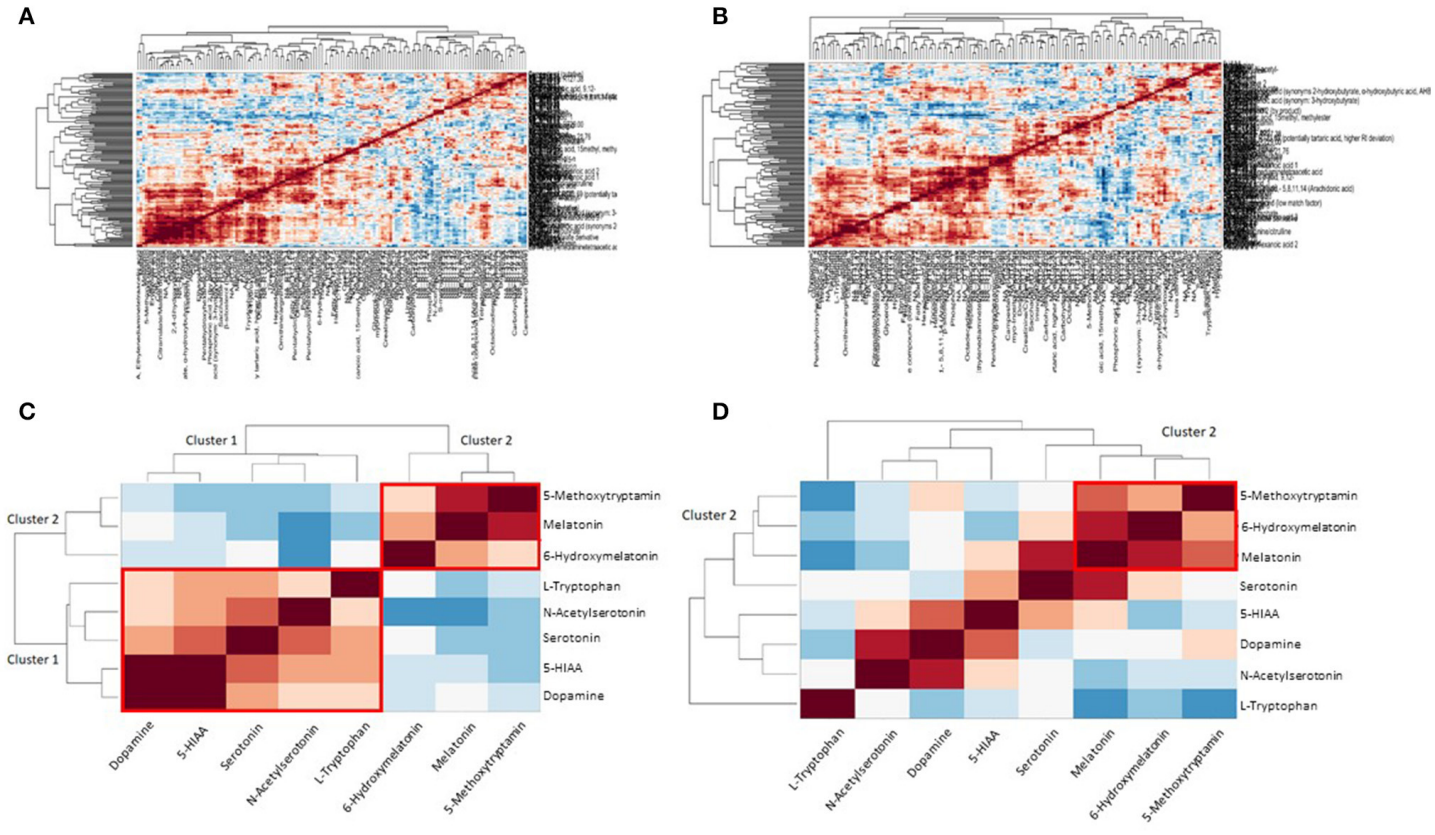
Metabolite correlation network analysis visualized as clustered correlation coefficient matrix. **(A)** Clustered heat map shows a significant metabolic signature of control samples. **(B)** GDM case show a different pattern compared to the control samples indicating a dramatic reprogramming of metabolism in GDM disease. **(C)** Detailed view of serotonin/melatonin metabolites in urine samples of control. Cluster 1 is a highly correlated serotonin cluster and cluster 2 a highly correlated melatonin cluster in normal metabolic conditions. **(D)** Detailed view of serotonin/melatonin metabolites in urine samples of GDM case. Cluster 1 disappeared in GDM cases and cluster 2 is conserved from control to GDM.

In Figures 3A,B clustering of correlation coefficients between all metabolites from plasma and from urine samples is shown. The clustered correlation map is very different between control and GDM cases indicating a large reprogramming of metabolism in GDM patients. This pattern can be interpreted as a signature of GDM. Details are discussed below and in the discussion. In Figures 3C,D detailed clustered correlation coefficient maps of the serotonin-related metabolites in urine samples are depicted. Here, a remarkable difference is the clustering of serotonin, dopamine, 5-HIAA, N-acetyl-serotonin and tryptophan (cluster 1) and 5-methoxytryptamin, melatonin and 6-hydroxy-melatonin (cluster 2) in the control samples whereas cluster 1 is completely dissolved and cluster 2 is maintained in GDM cases (see Figures 3C,D).

**Figure 4:**
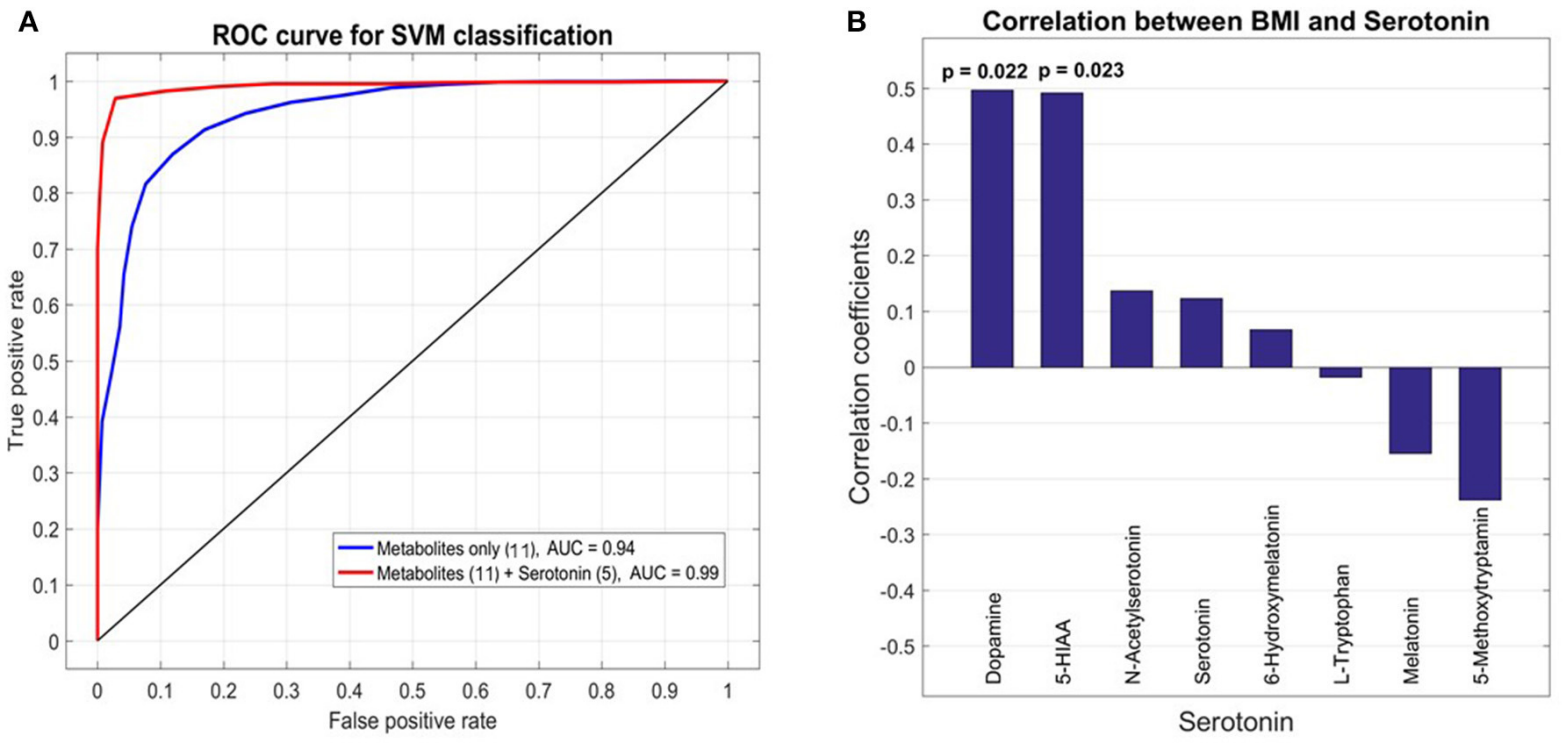
Receiver operating characteristic (ROC) analysis of selected metabolites from plasma and urine samples of GDM vs. control patients. **(A)** Area under curve (AUC) for selected metabolites from plasma analysis and combined analysis of selected analysis of plasma and urine analysis. The final AUC is 0.99. Selection of variables was performed by LASSO regression (see section Materials and Methods). 11 Metabolites from blood samples and 5 metabolites from urine samples were selected (for further information see results and discussion). **(B)** Correlation analysis of serotonin, associated metabolic intermediates and dopamine measured in urine with BMI. The strongest correlation is found for dopamine.

To select for the set of most discriminatory variables from blood and urine sample metabolites a LASSO (least absolute shrinkage and selection operator) regression method was employed. For the 21 patients (10 GDM, 11 nGDM) with serotonin and metabolic intermediate measurements, we built statistical models to predict GDM from a combination of 131 plasma metabolites as well as 8 urine metabolites related to serotonin metabolism. LASSO regression wrapped with a linear SVM (support vector machine) classifier, was applied to select the best subset of variables that achieves the highest prediction accuracy. We found that the best subset of metabolites plus BMI achieves a good prediction performance with an AUC (Area under curve) value 0.94 of the ROC (Receiver operating characteristic) curve. These metabolites are glycolic acid, urea, methionine, erythronic acid, an unknown organic acid (potentially tartaric acid), glutamine, an unknown carbohydrate, unknown, alanin, serin and tryptophan. Next, we tested whether the prediction accuracy can be improved by combining the serotonin data from urine analysis. Following the same procedure, the best model adds five serotonin compounds, namely, serotonin, 5-HIAA, L-tryptophan, melatonin and 6-hydroxymelatonin, and achieves a higher AUC value of 0.99. The comparison between these two models is shown in Figure 4A.

### Correlation between BMI and metabolites of serotonin metabolism in urine samples

Pearson correlation coefficients (PCC) were calculated between 8 urine target compounds and BMI (Figure 4B). Significant associations were found between BMI and dopamine (PCC = 0.50, *p*-value = 0.022), and 5-HIAA (PCC = 0.49, *p*-value = 0.023), respectively.

## Discussion

### Changes in serotonin metabolism in GDM vs. control group

L-tryptophan and its metabolites serotonin (5HT) and melatonin have notable functions in regulating growth and development of the fetus (Glover, 2015; St-Pierre et al., 2016; Wu et al., 2016) and are involved in a magnifique number of physiological pathways and adaption processes during pregnancy (Sano et al., 2016). As L-trytophan (TRP) is highest ranked in the PLS-analysis (Figure 1D), we searched for differences regarding serotonin metabolism. Therefore, we applied a serotonin assay by using stable isotope dilution direct electrospray ionization mass spectrometry (SID-MS, see section Materials and Methods) to analyze urine samples of the same patients. In the measurements the control and the GDM patients showed significantly different concentrations of serotonin and intermediates of this pathway in urine samples (see Figure 2): serotonin levels were higher in women with GDM compared with women without GDM. Besides the well-known role of serotonin in mood and feeding behavior, recent results renewed the interest in the role of serotonin in metabolic diseases (Almaça et al., 2016). Conditions of metabolic demand lead to the production of serotonin in the pancreatic islet (Kim et al., 2010; Goyvaerts et al., 2015, 2016). Further, studies showed that serotonin via autocrine signaling increases beta- cell function and ß-cell mass during insulin resistant states (e.g., pregnancy). In mouse models evidence suggests that increased serotonin in islets during pregnancy drives ß-cell expansion (Kim et al., 2010). Additionally, serotonin levels were shown to rise in regard to BMI (Almaça et al., 2016). A recent study showed that ß-cell-derived serotonin inhibits glucagon secretion, a hormone which elevates the blood glucose levels. Based on this, our results could be explained by (1) a different lifestyle (nutrition: protein intake) of the patients with GDM and those without GDM (2) a higher BMI of women with GDM compared to the control group could be a reason either, or (3) higher serotonin levels in the case of disrupted metabolism, like GDM, could substantiate that serotonin is a factor in maintaining normoglycemia, the increase presenting a possible compensatory mechanism.

In summary, changes in L-tryptophan in the GDM group pointed to an altered serotonin metabolism which indeed is changed in GDM patients. Accordingly, alterations in serotonin metabolism may be fundamental in the pathogenesis of GDM. Nevertheless, such findings need to be confirmed by further studies analyzing larger cohorts.

### Glucose-L-alanine cycle

Some of the significantly changed metabolites are found in the glucose-L-alanine cycle, which plays a role in glycolysis and gluconeogenesis. These findings appear to be relevant for the energy metabolism in GDM. The glucose-alanine cycle is a biochemical route between muscle and liver metabolism. In the muscle, protein is degraded resulting in glutamic acid. Subsequently, the alanine aminotransferase transfers the amino group from glutamate to pyruvate, which arises from glycolysis. L-alanine is formed and then transferred from muscle to the liver through the blood. Finally, the amino acid is used in the liver for gluconeogenesis. Several other metabolites show an alteration in concentrations from the GDM group (see Supplementary Figure S1). Furthermore, we analysed also the alanine-amino-transferase activity and identified a significant change between the case and control groups confirming the metabolomics data (data not shown). A recent study suggests an up-regulated glycolysis and higher alanine concentration in T2DM patients (Huang and Joseph, 2012). Like in our study another recent study discovered increased concentrations of alanine and lactate as potential gluconeogenic precursors (Galazis et al., 2012) as well as alanine and glutamate as significant marker in GDM (Bentley-Lewis et al., 2015).

### Strong markers of GDM disease −2- and 3-hydroxybutanoic acid

Ketone bodies play a role in the fatty acid biosynthesis. 3-hydroxybutanoic acid (β-hydroxybutyrate; BHBA) is an organic acid, which is used for the biosynthesis of fatty acids. BHBA reached statistical significance between the case and control group. Furthermore, 2-hydroxybutanoic acid (α-hydroxybutyrate; AHBA) shows the lowest *p*-value of all metabolites between control and GDM and therefore is one of the strongest metabolic alterations (Supplementary Figure S1).

Our results confirm already well documented conclusions: AHBA is suggested as an early biomarker for insulin resistance and proposed to identify insulin resistance earlier than current diagnostic tests (Gall et al., 2010). Previous research has studied liver and plasma metabolome in mice to identify early alterations in development of insulin resistance and found significant changes in metabolites, of which AHBA is also significant in our study (Li et al., 2009). Altogether AHBA tends to be important for decreased insulin sensitivity in GDM and may be critical in the prevention and treatment of diabetes as potent biomarker.

### Propanoate metabolism

In consideration of four metabolites—lactic acid, AHBA, beta-alanin, and valine—of the propanoate pathway which show a significant change in case and control (see Supplementary Table S1 and Supplementary Figure S1) it seems reasonable to propose that the whole propanoate pathway is changed in the case group. On the basis of our data we suggest an adaption in the propanoate metabolism as described in an *in-vivo* study for DM by Huang in 2006 (Huang et al., 2006). Literature-derived evidence comprises a lipidomic analysis which found statistically significant pathways, among them the propanoate pathway (Zhao et al., 2013).

### Degradation of valine and fatty acids

Among the amino acids especially valine is one with the highest significance. Further, intermediates of the citric cycle are significantly changed in GDM (see Supplementary Figure S1). It seems very likely that the rate of degradation of amino acids is varying between case and control group. From protein breakdown L-valine is generated and finally succinyl-CoA appears to contribute to the citric acid cycle in muscle. In liver succinyl-CoA can be used for synthesis of glucose. Therefore, there is considerable evidence of an alteration in gluconeogenesis rates in the pathogenesis of GDM. 2,4-dihydroxybutyrate is described in the HMDB as usually absent in normal human urine extracts or present only in trace amounts in neonates (Wishart et al., 2013). However, in our study this metabolite was identified as being significantly changed between case and control group in blood samples.

### A metabolic signature of GDM by combined plasma and urine metabolomics analysis

The analysis of metabolite correlation networks is a powerful method for the description of systemic biochemical regulation (Steuer et al., 2003; Weckwerth, 2003, 2011; Weckwerth et al., 2004a; Weckwerth and Morgenthal, 2005; Nägele et al., 2014). Recently, we demonstrated that differential metabolite correlation or covariance networks reflect biochemical regulation depending on the genotype or other factors (Weckwerth et al., 2004a; Sun and Weckwerth, 2012; Nägele et al., 2014). To reveal a picture of the metabolite correlation network in control vs. GDM case samples we used here a clustered correlation coefficient matrix visualization (Figure 3). Based on this visualization clearly distinguishable metabolite signatures differentiate control and GDM cases (Figures 3A,B). In a more detailed analysis of serotonin metabolites in urine samples a highly significant reprogramming of serotonin/melatonin metabolism is visible. Whereas serotonin and tryptophan and related metabolites form a highly correlated cluster (cluster 1 in Figure 3C) this cluster is compromised in GDM metabolism. In contrast, the melatonin cluster (cluster 2 in Figures 3C,D) is conserved. There are several reports that serotonin metabolism and especially serotonin transport is impaired in GDM (Li et al., 2014). Subsequently this would lead to changes in biochemical correlation networks as shown in our study. Further studies are necessary to underline the dynamics of these different metabolic signatures and especially reveal causal relationships between changed metabolite levels and enzymatic regulation (Sun and Weckwerth, 2012; Nägele et al., 2014).

To select for the best set of predictive candidate biomarker we used LASSO regression (see sections Materials and Methods and Results). The combined analysis of plasma metabolomics and targeted metabolomics of serotonin metabolism in urine revealed an improvement in prediction accuracy for GDM (see Figure 4A). Recently, a study of type 2 diabetes (T2DM) showed no improvement of prediction accuracy by integrating NMR metabolite profiling of blood and urine samples (Friedrich et al., 2015). In contrast, our targeted approach of profiling specific serotonin-related metabolites in urine indeed improved the prediction accuracy in GDM. Thereby the specificity of serotonin metabolism in GDM is supported.

Integrative regression analysis revealed further interesting metabolite markers. Glycolate was also recently detected as a T2DM metabolite marker in woman (Friedrich et al., 2015). In another study on T1D in rats urea was found to be a significant marker in plasma samples (Zhang et al., 2008). Several amino acids are part of the identified metabolic signature for GDM such as methionine, glutamine, alanine, serine, and tryptophan. Tryptophan is directly related to serotonin metabolism. Consequently, the extension of the metabolic signature by metabolites derived from the serotonin pathway improved the prediction accuracy (Figure 3A). Another very interesting metabolic marker is erythronic acid. This acid is a side-product from the degradation of so called advanced glycation end-products (AGE) such as fructosamine (Jakus and Rietbrock, 2004). AGE's play an important role in the pathophysiology of diabetes. We further correlated serotonin-related metabolites and others measured in the urine samples with the BMI of the cohort and found a strong correlation with dopamine. An intimate relationship between dopamine and obesity is postulated in the “reward deficiency syndrome” (Blum et al., 2014). We will investigate this relationship in more detail in future studies.

## Conclusion

Plasma metabolomics is a comprehensive technology for the rapid and in depth analysis of all kinds of metabolic diseases. The amount of information by this approach is still not fully understood. Here, we applied this technology for the early diagnosis of GDM. Based on the physiological interpretation of identified metabolic markers we developed the hypothesis that serotonin is involved in GDM pathogenesis. We tested this hypothesis by targeted profiling of serotonin-derived metabolites in urine samples and the integration of plasma and urine metabolic markers improved the prediction accuracy of GDM in our study. This workflow reveals the power of metabolomics screening of metabolic diseases, especially in the context of physiological interpretation of identified significant changes in metabolites (McCabe and Perng, 2017). In future studies, we will test our hypothesis in more detail and will extend the study to larger cohorts to unambiguously validate or falsify the potential metabolic marker from our study. Furthermore, our study lays the ground to investigate GDM in more detail on a biochemical and physiological basis.

## Author contributions

WW and AK-W conceived the study. SD, LF, ML, NH, ST, and HD performed the experiments. SD, LF, NH, HD, XS, GB, and WW analyzed the data and performed statistics. WW, WJ, KL, and AK-W provided the reagents, materials and analytical tools. WW and ML wrote the manuscript. All authors revised the manuscript and approved the final manuscript.

### Conflict of interest statement

The authors declare that the research was conducted in the absence of any commercial or financial relationships that could be construed as a potential conflict of interest.
