# Supplementary material for: Combined Metabolomic Analysis of Plasma and Urine Reveals AHBA, Tryptophan and Serotonin Metabolism as Potential Risk Factors in Gestational Diabetes Mellitus (GDM)
Source: Front Mol Biosci. 2017 Dec 21;4:84. doi: 10.3389/fmolb.2017.00084 (PMC5742855; doi:10.3389/fmolb.2017.00084)

## Slide 1
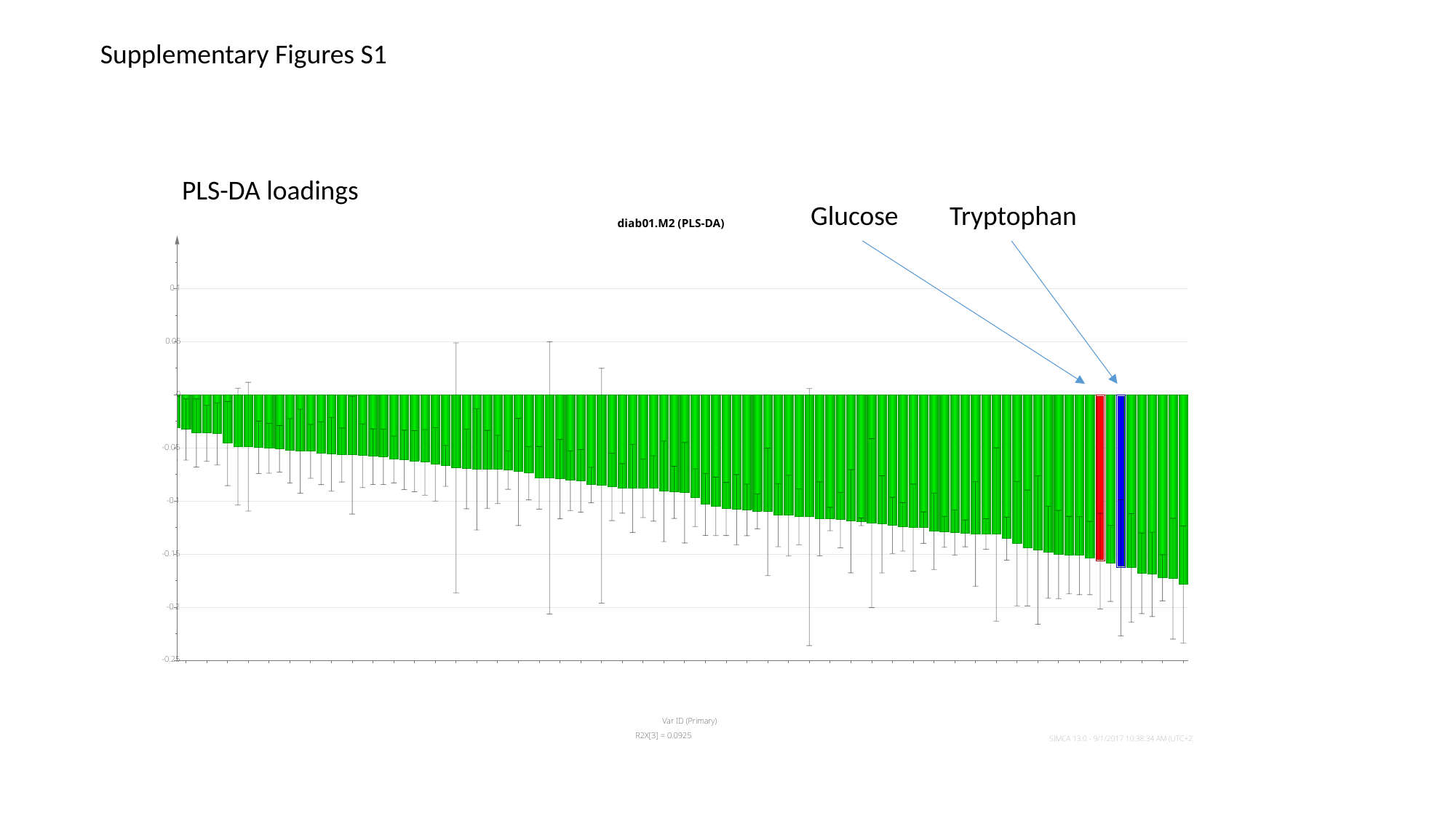

Supplementary Figures S1
PLS-DA loadings
Glucose
Tryptophan

## Slide 2
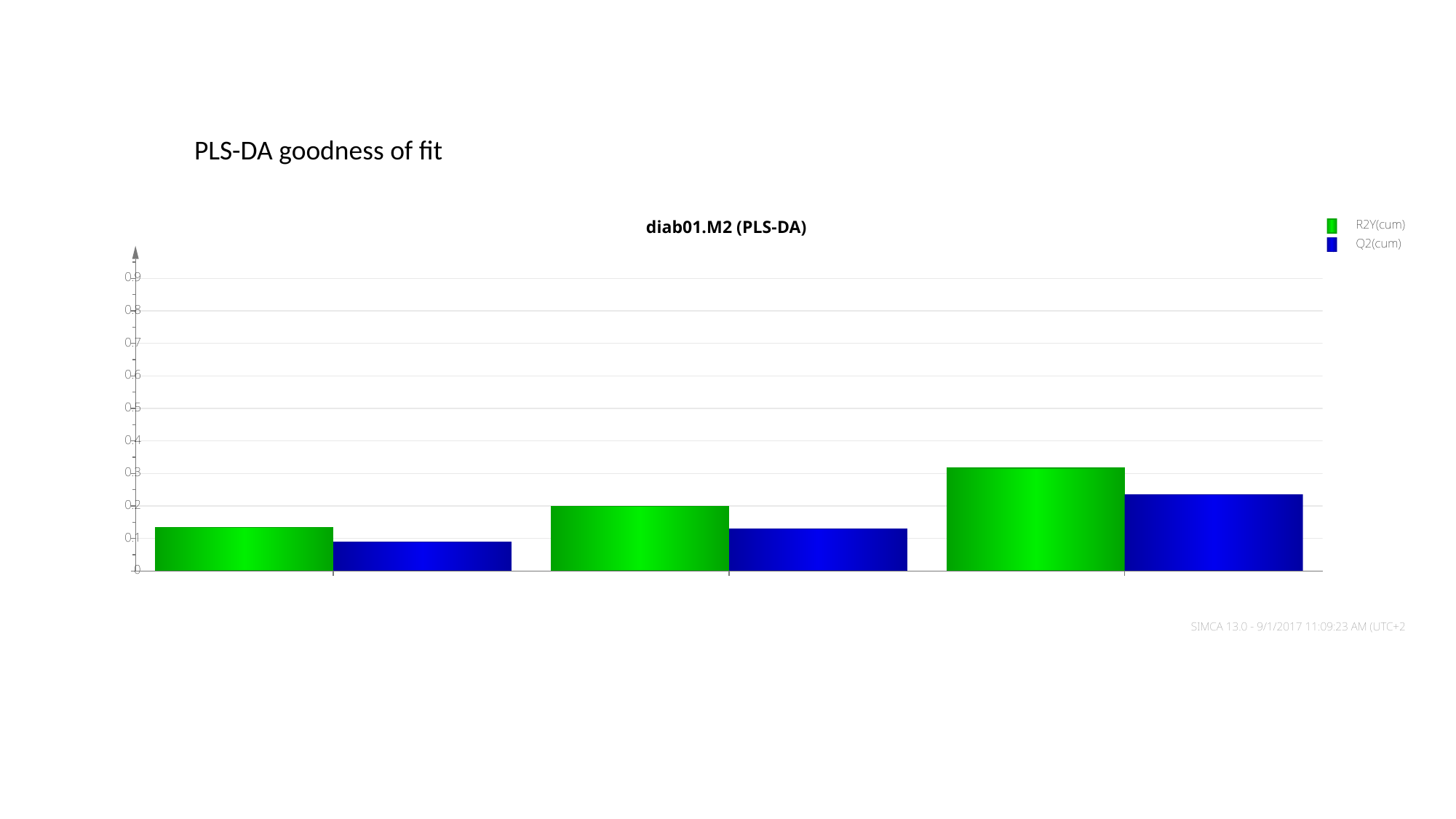

PLS-DA goodness of fit

## Slide 3
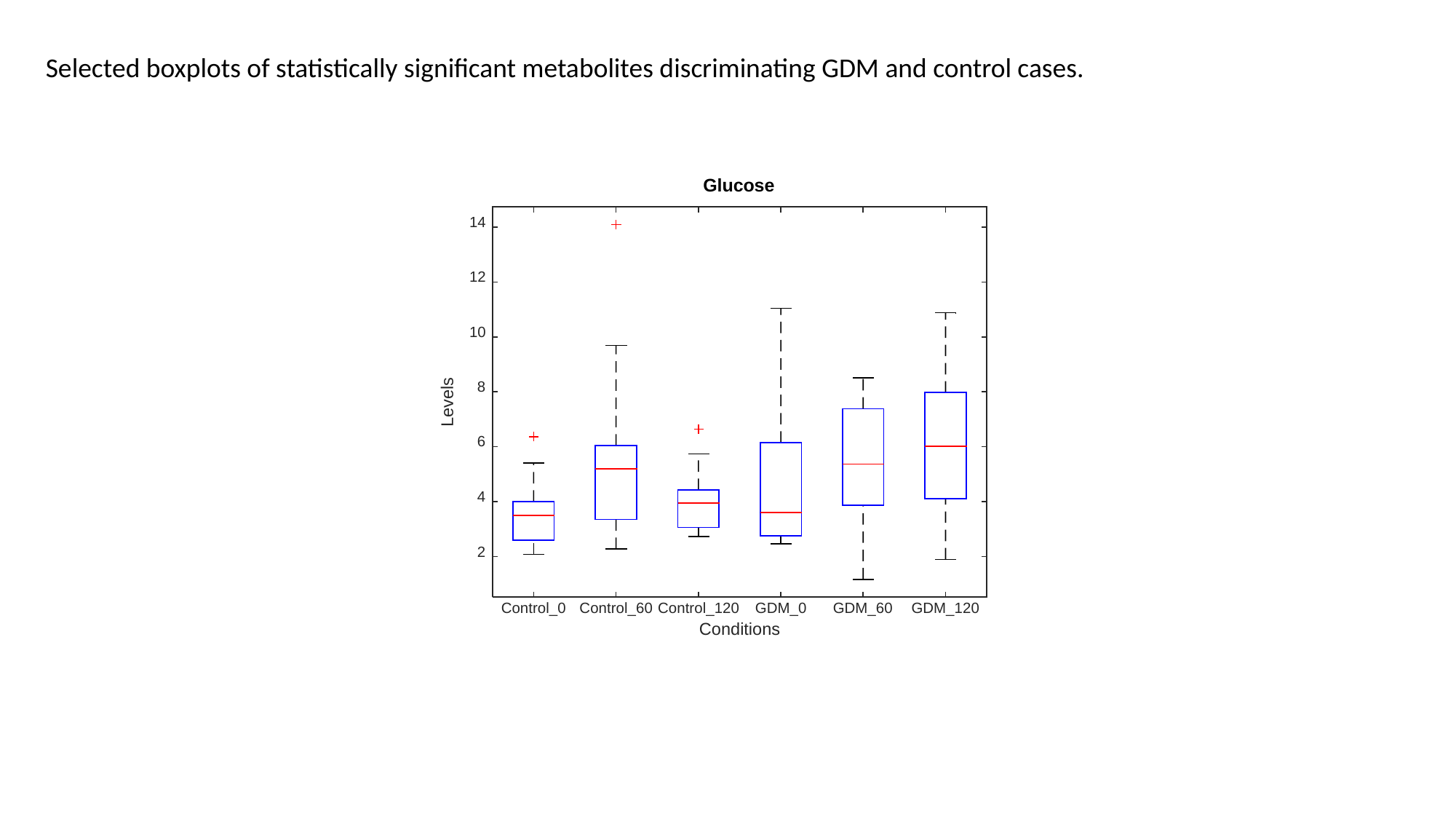

Selected boxplots of statistically significant metabolites discriminating GDM and control cases.

## Slide 4
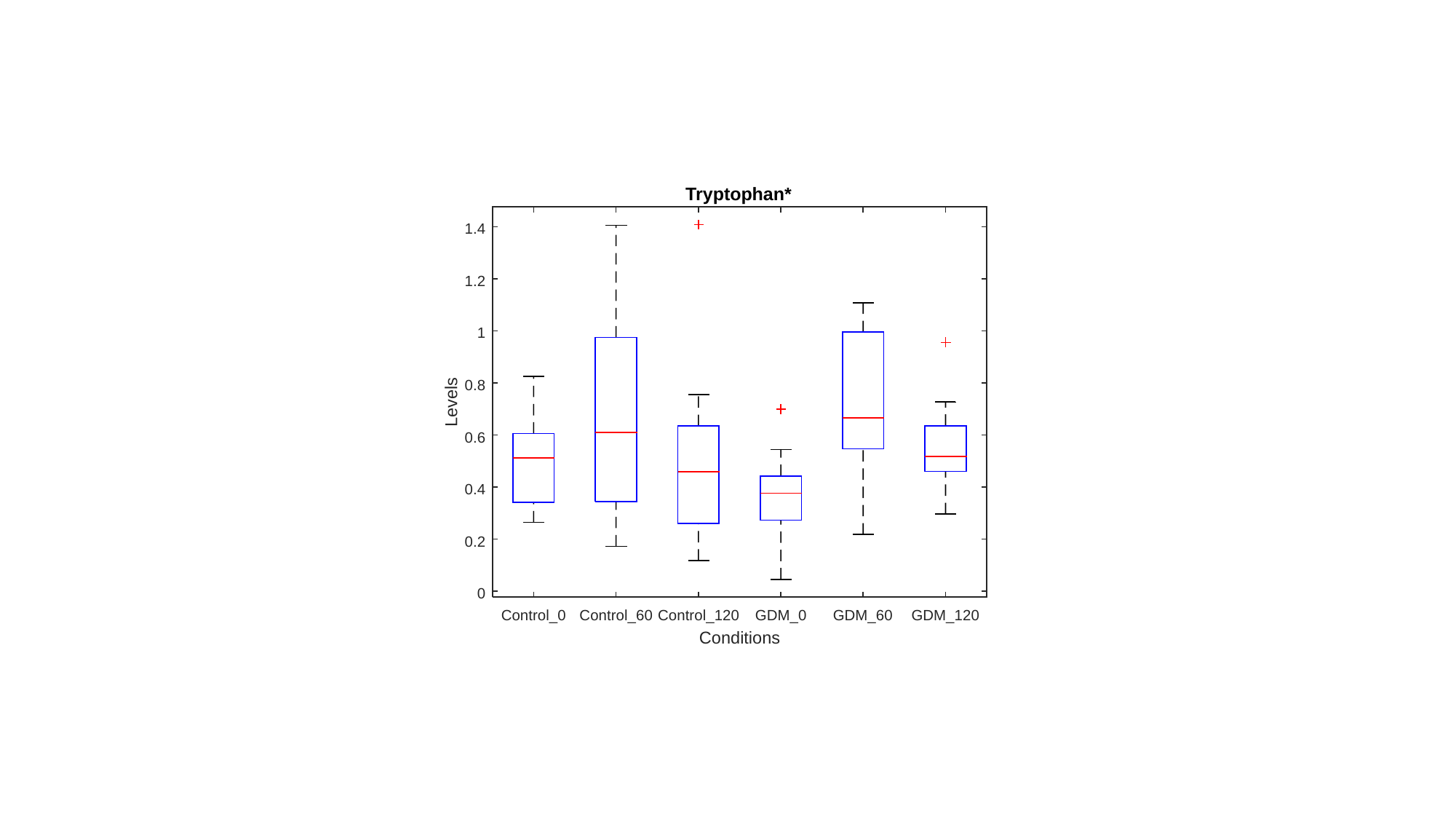

## Slide 5
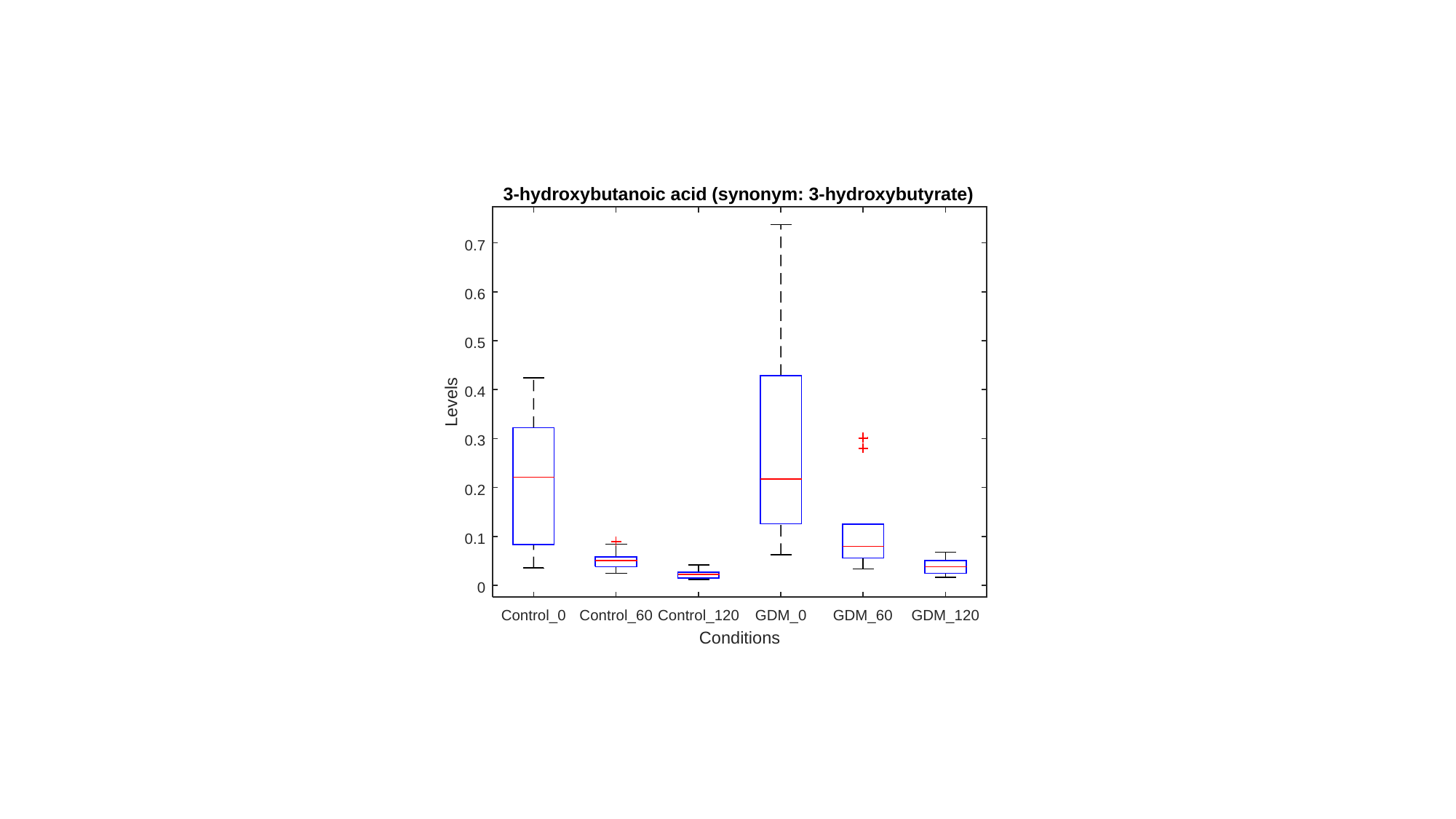

## Slide 6
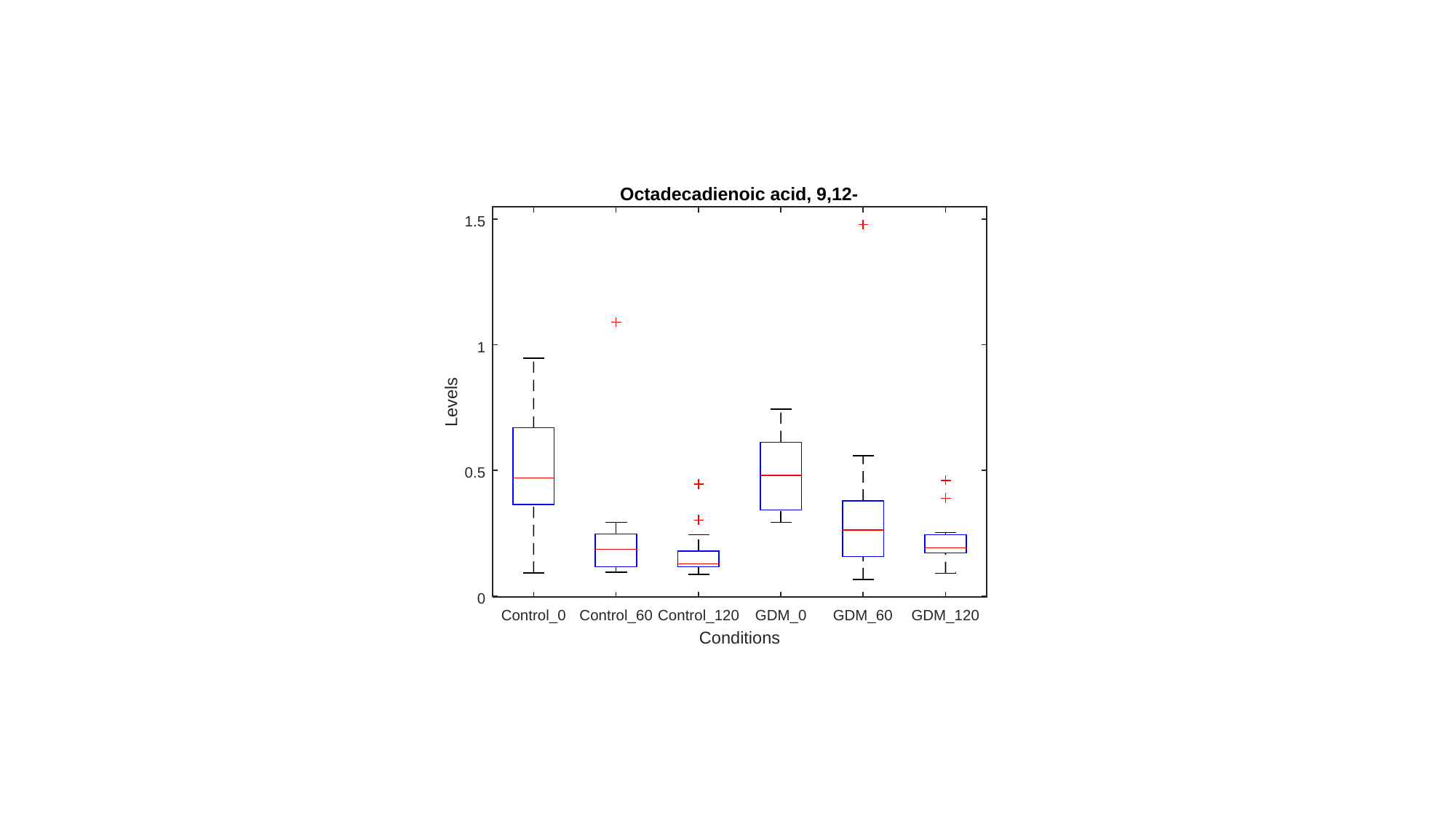

## Slide 7
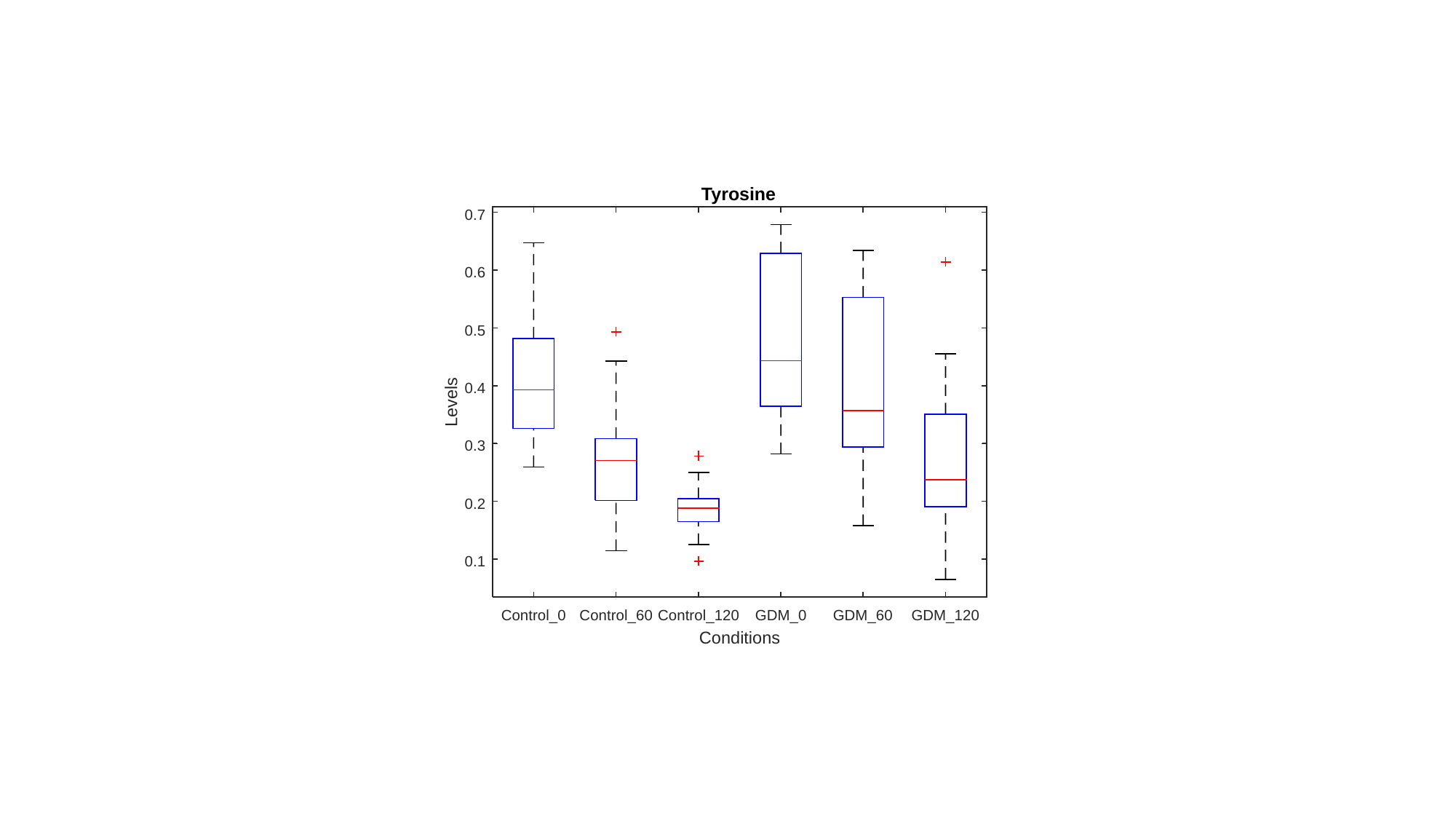

## Slide 8
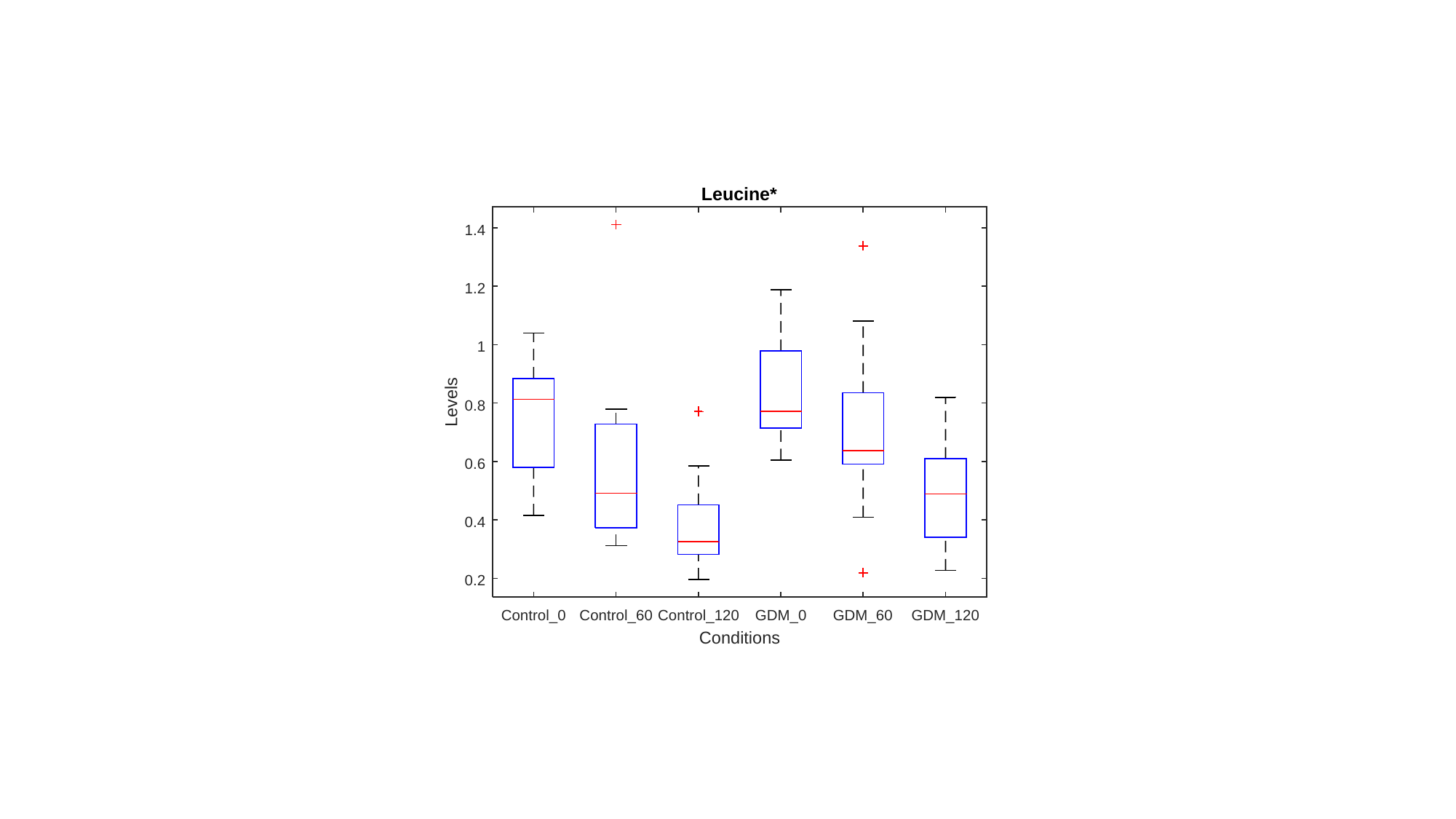

## Slide 9
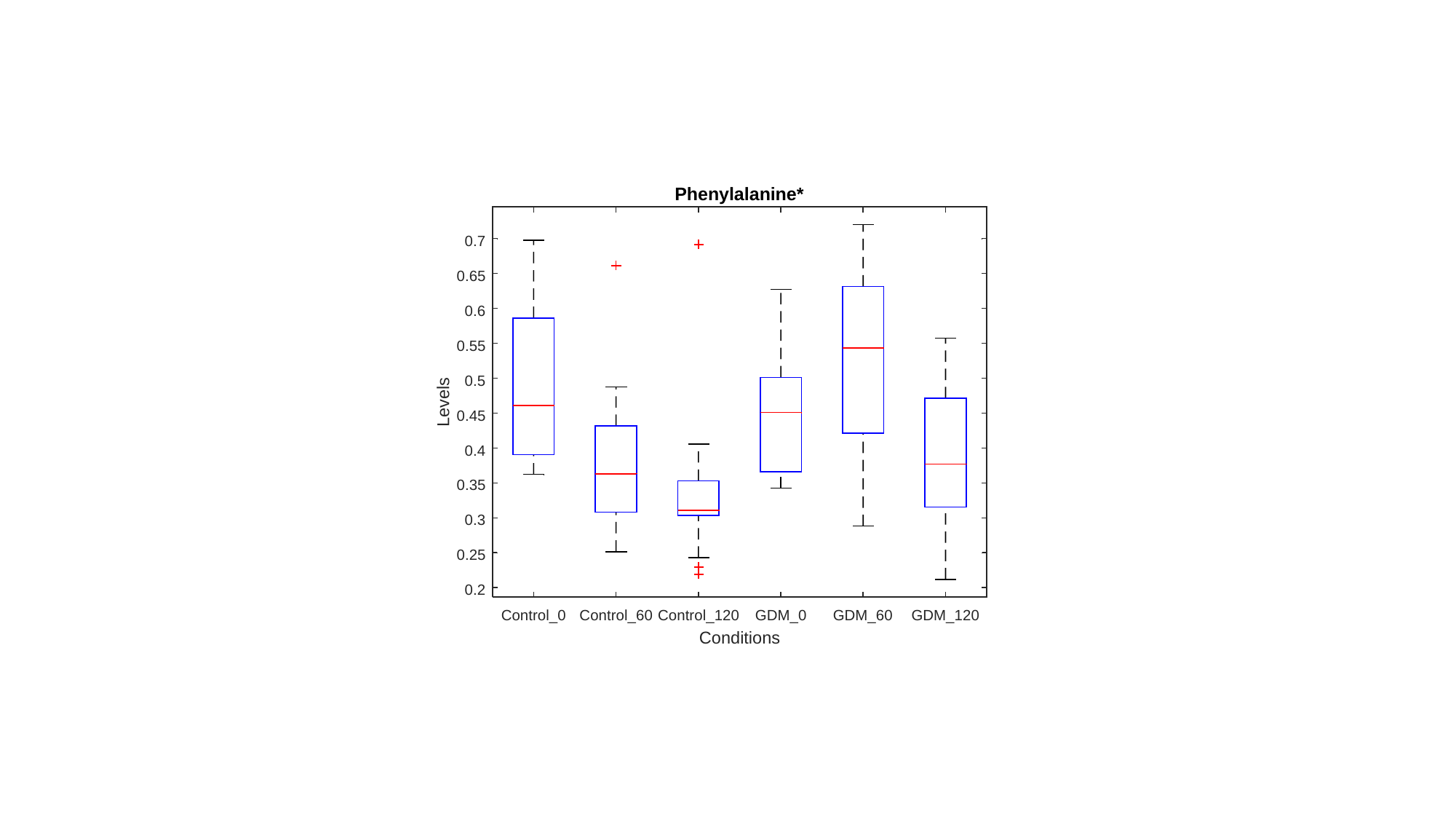

## Slide 10
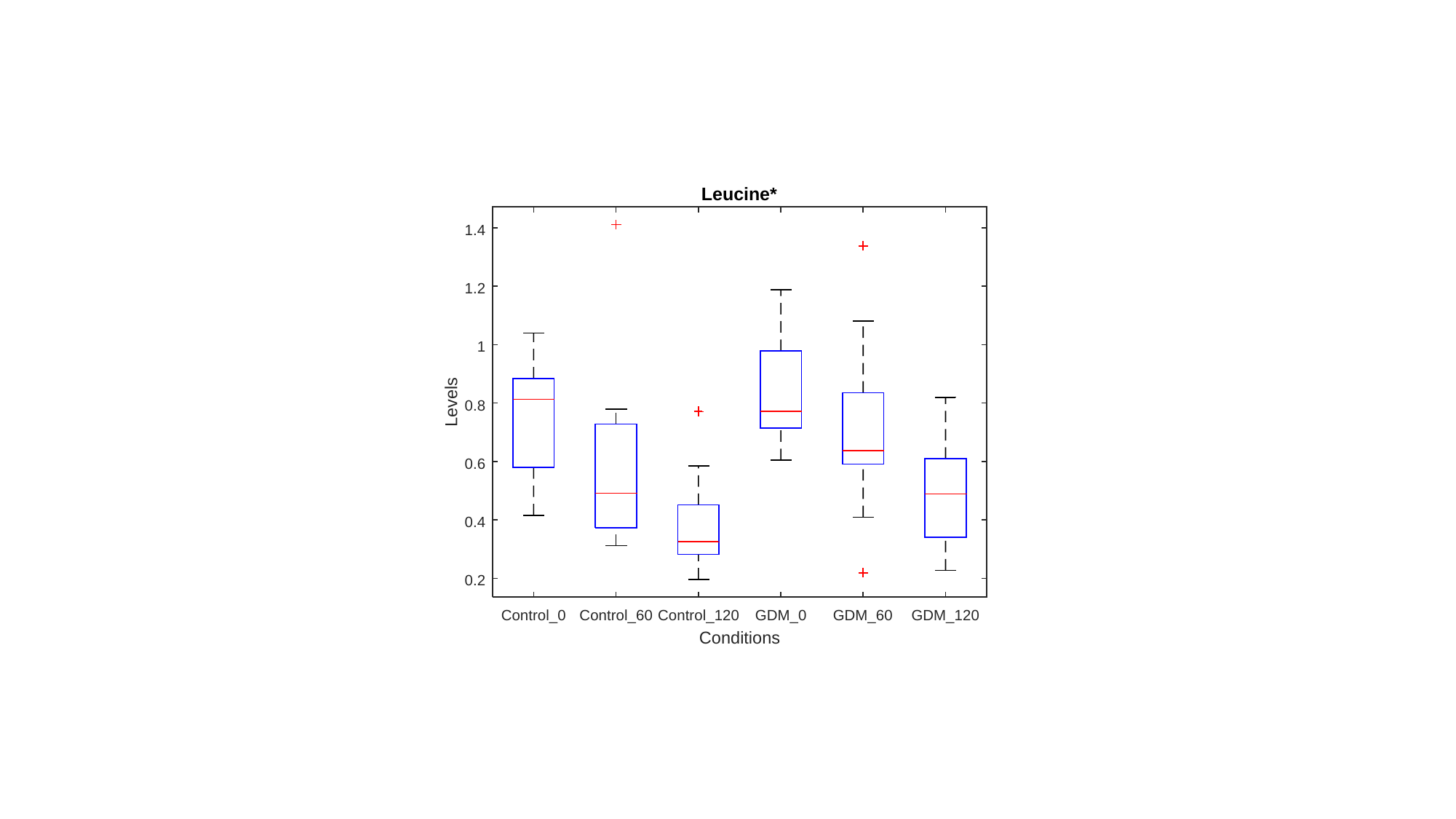

## Slide 11
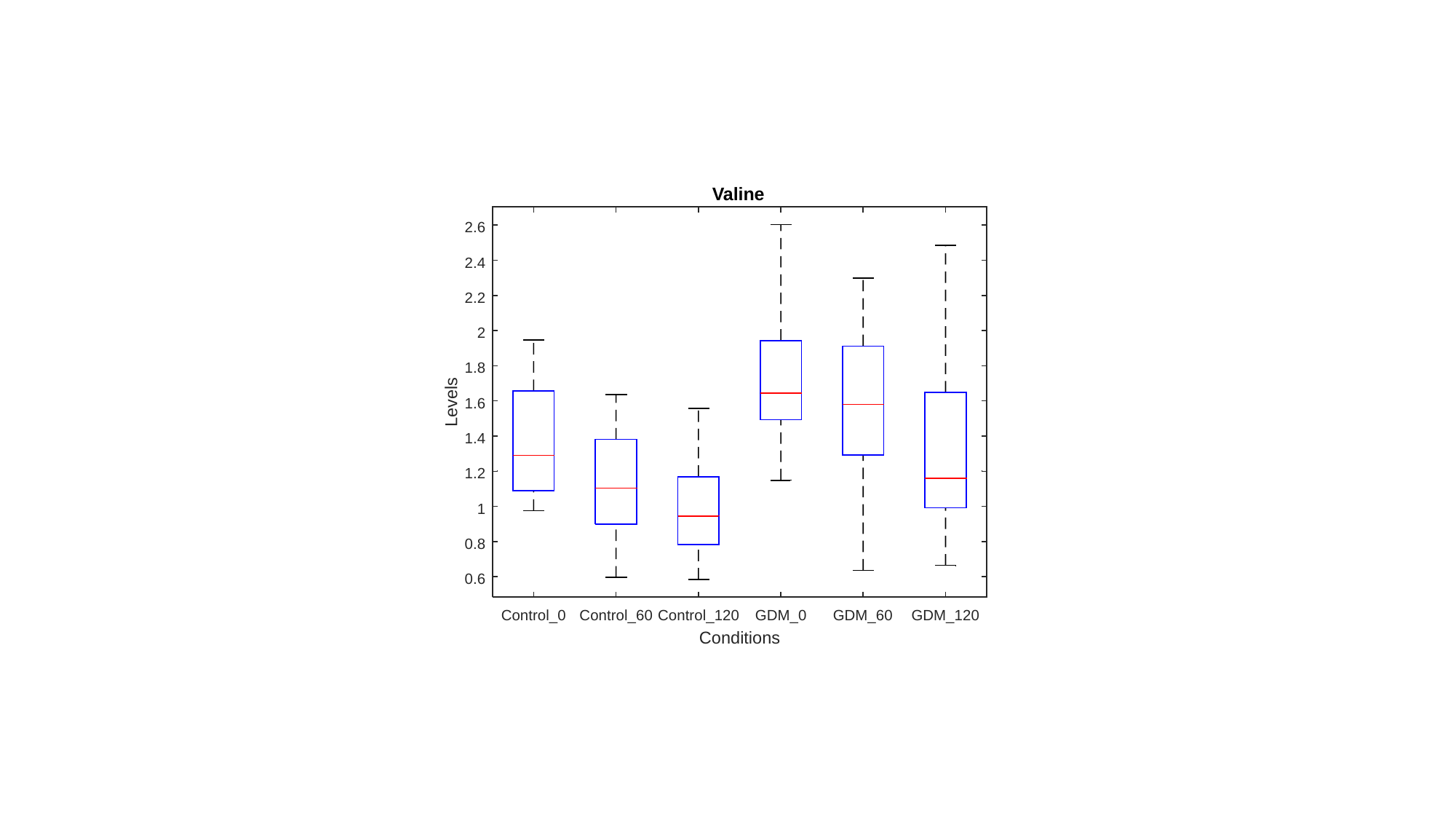

## Slide 12
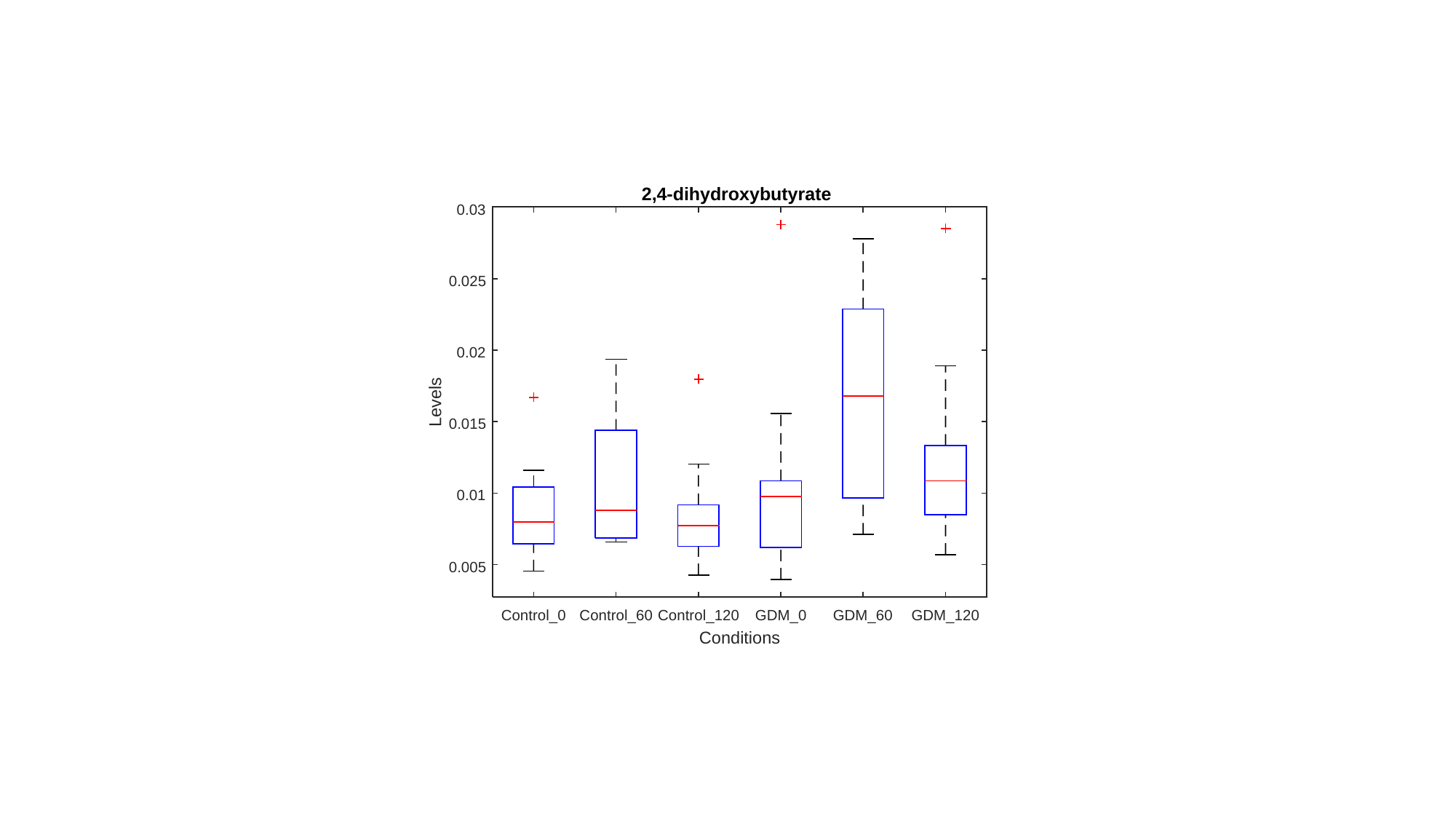

## Slide 13
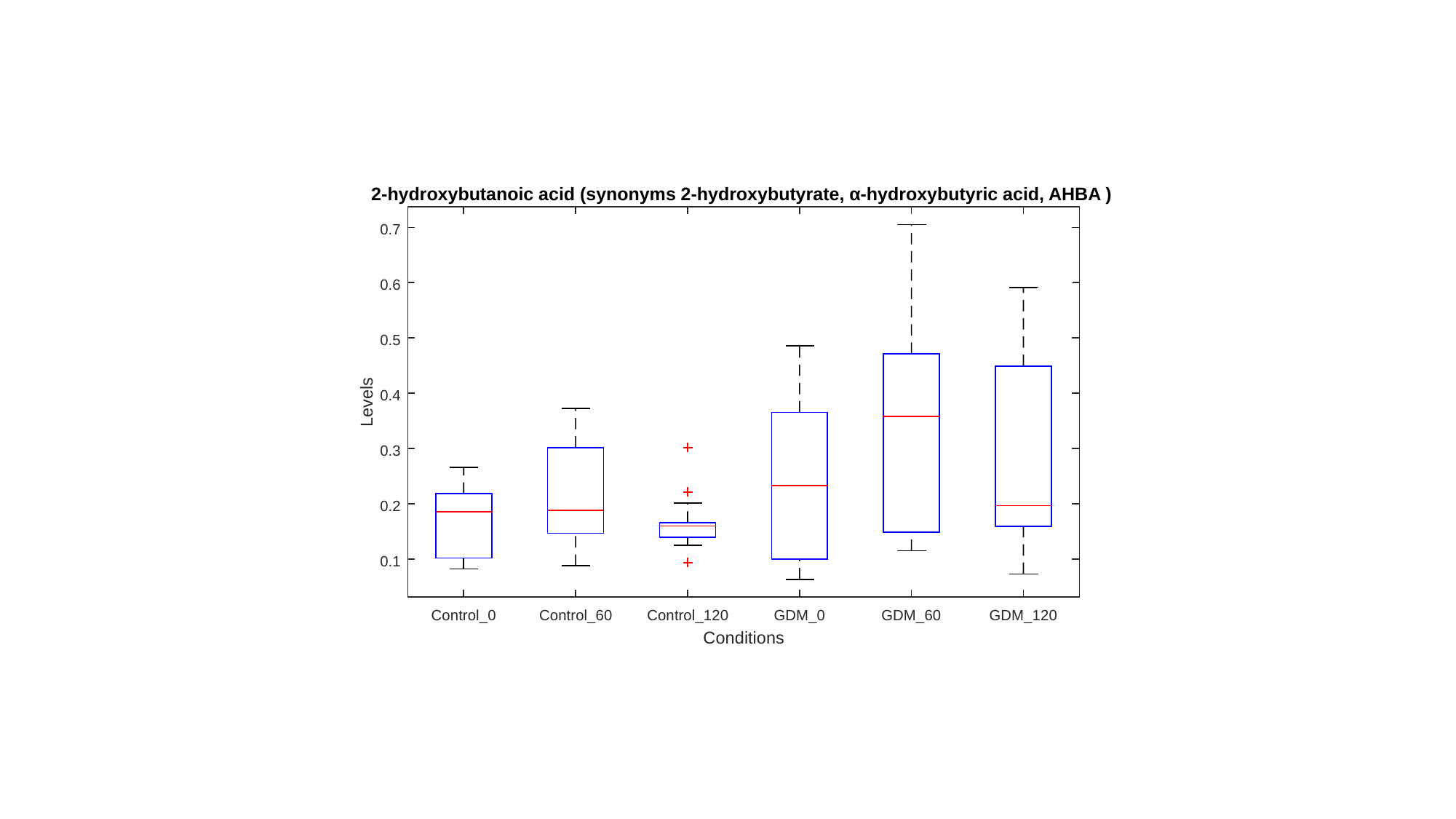

## Slide 14
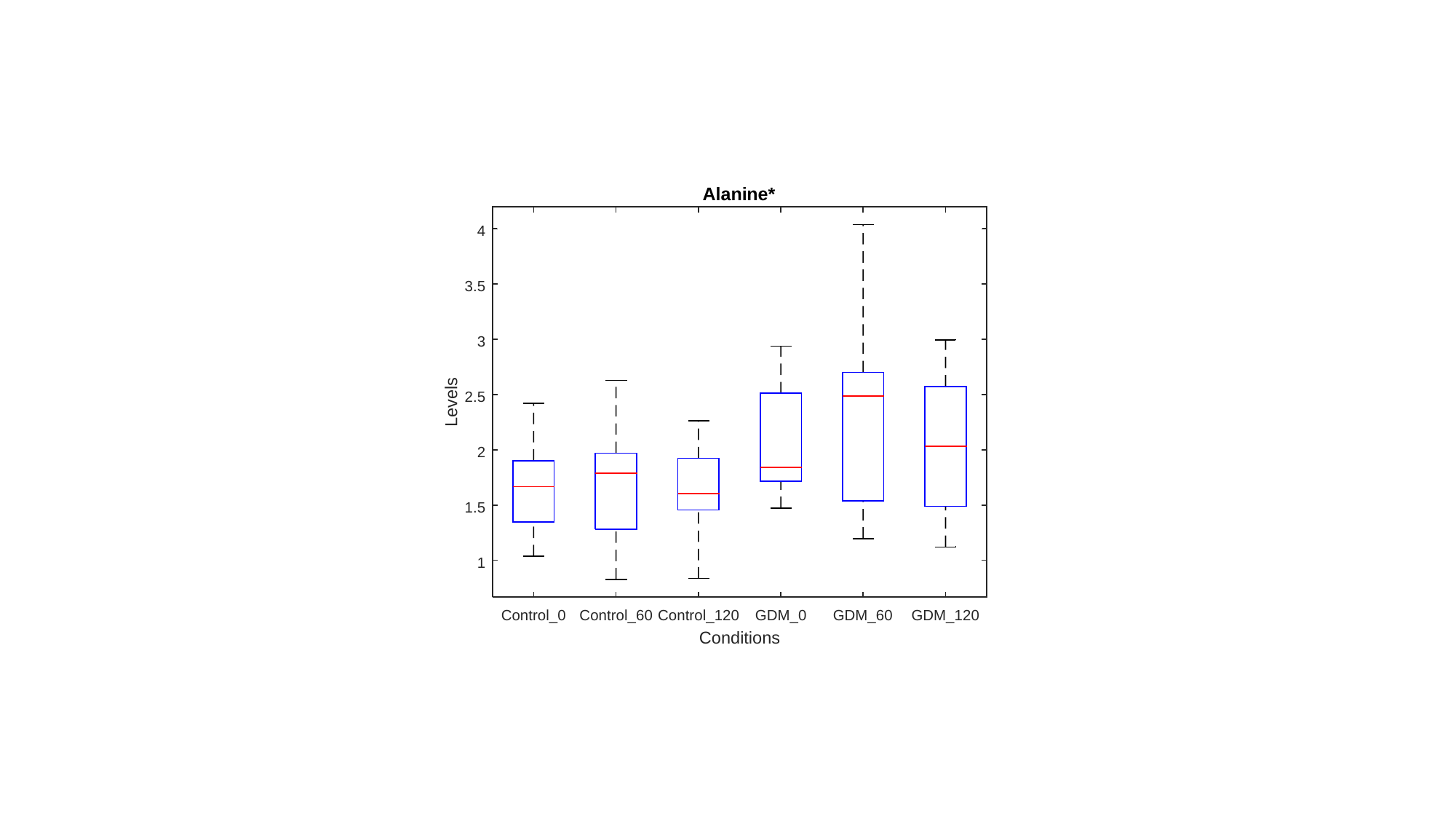

## Slide 15
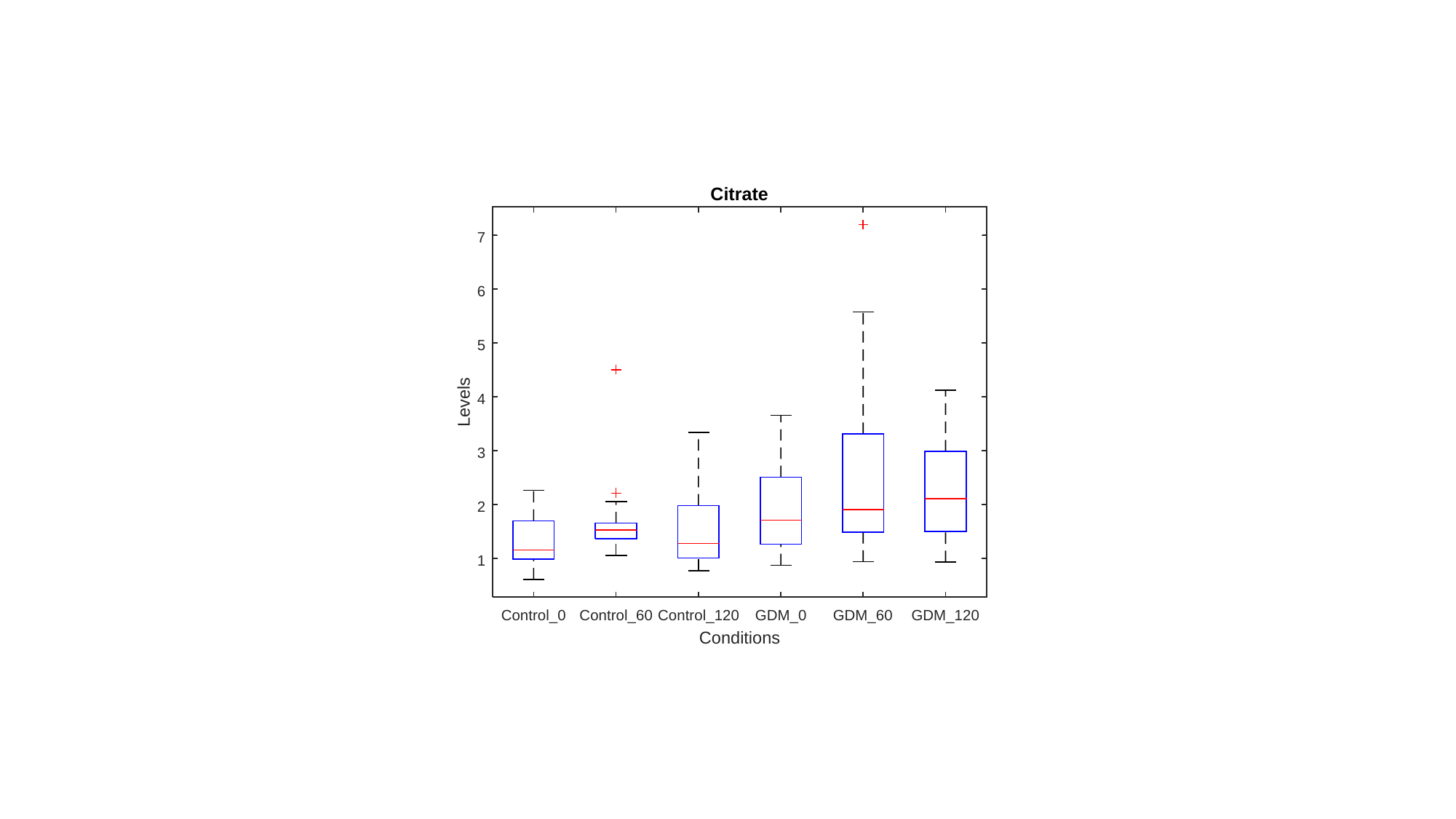

## Slide 16
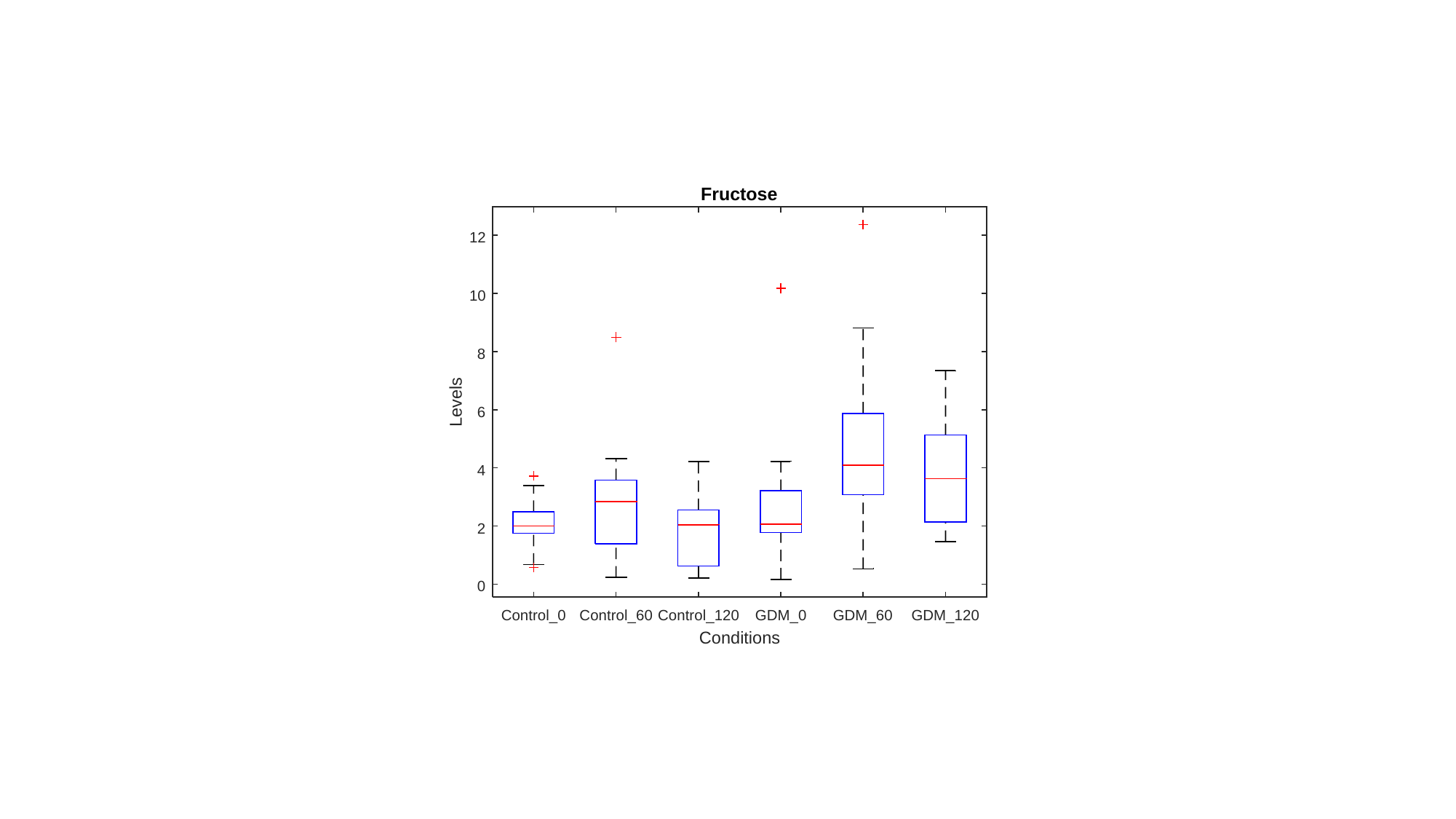

## Slide 17
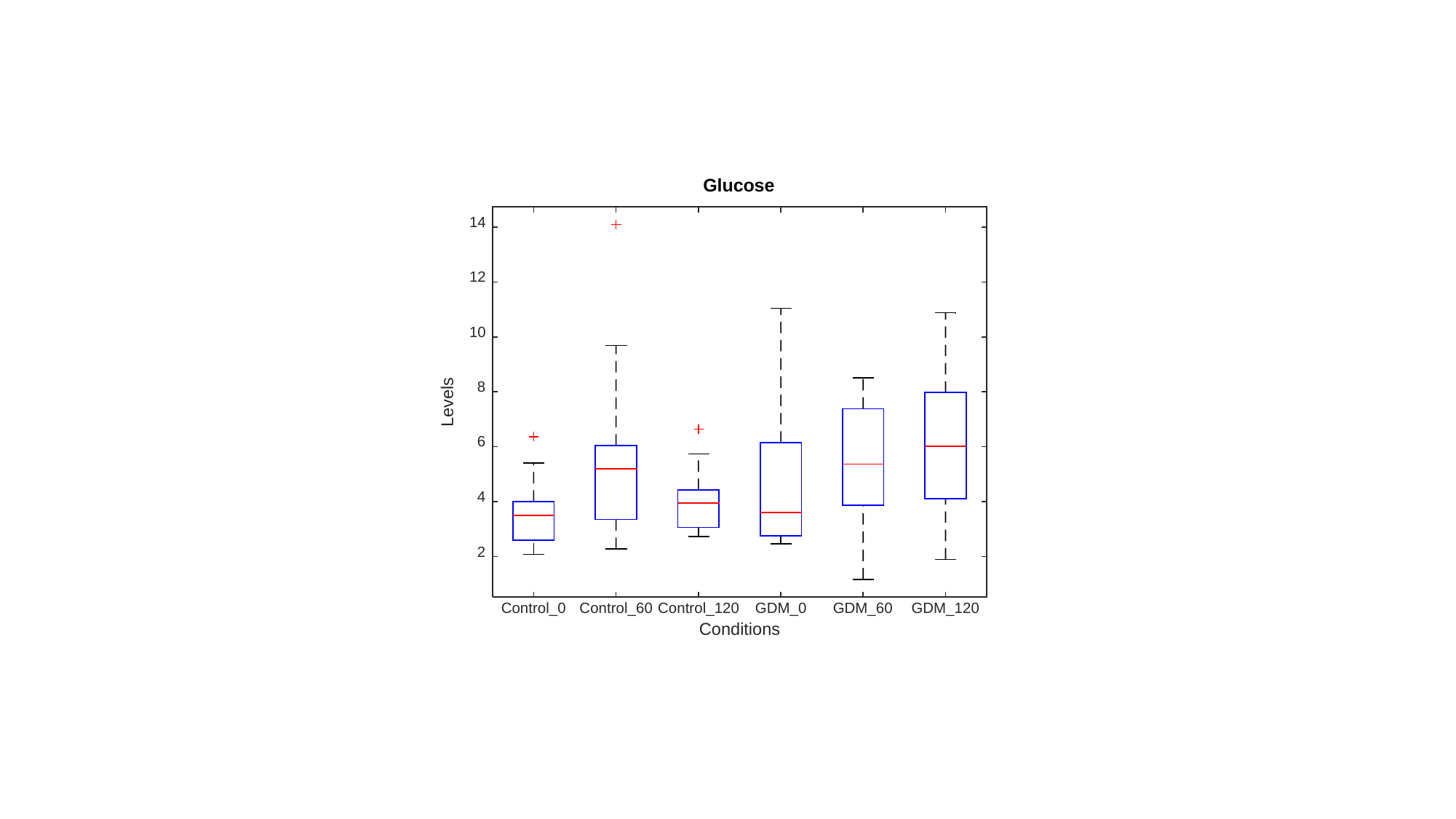

## Slide 18
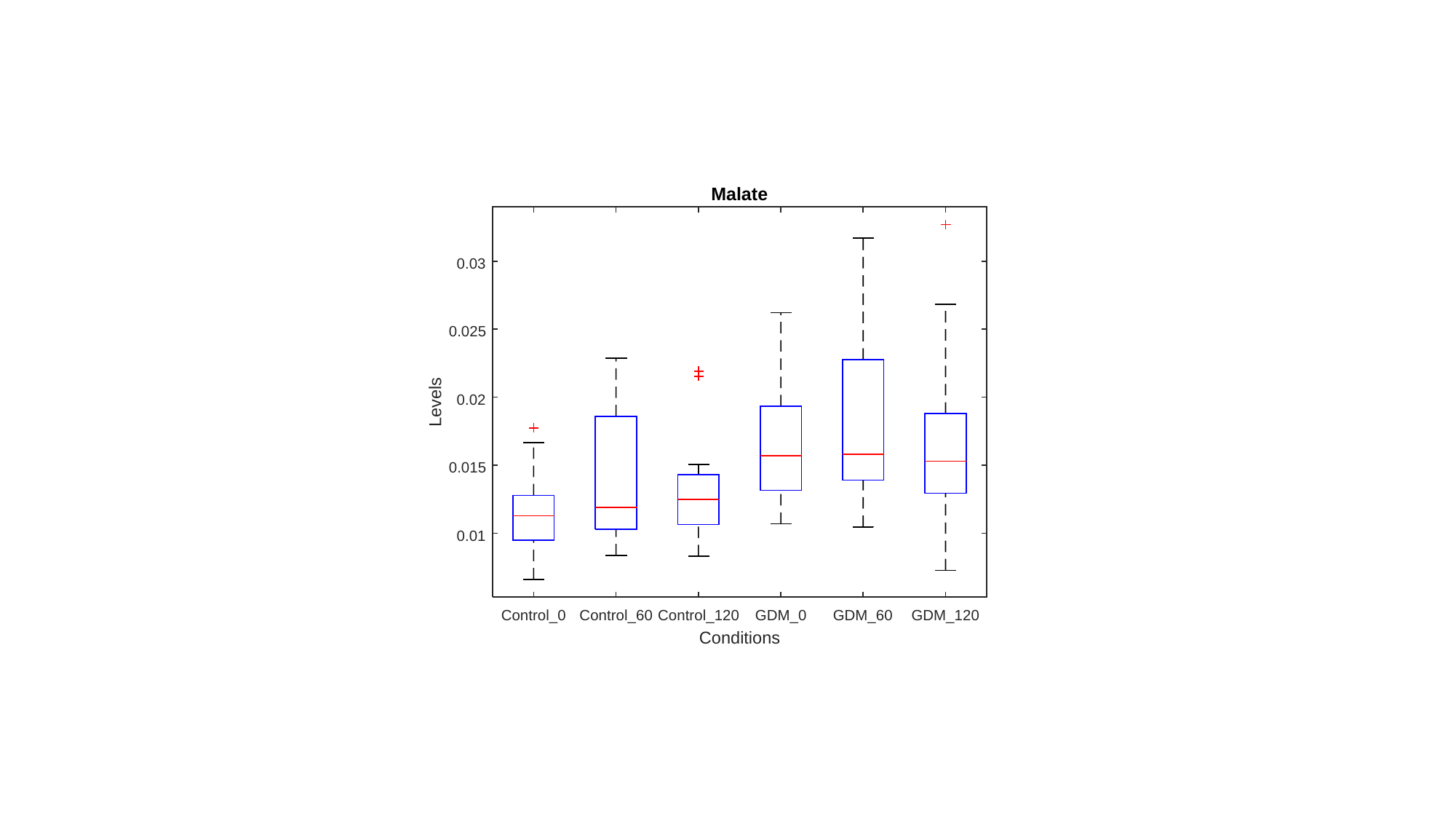

## Slide 19
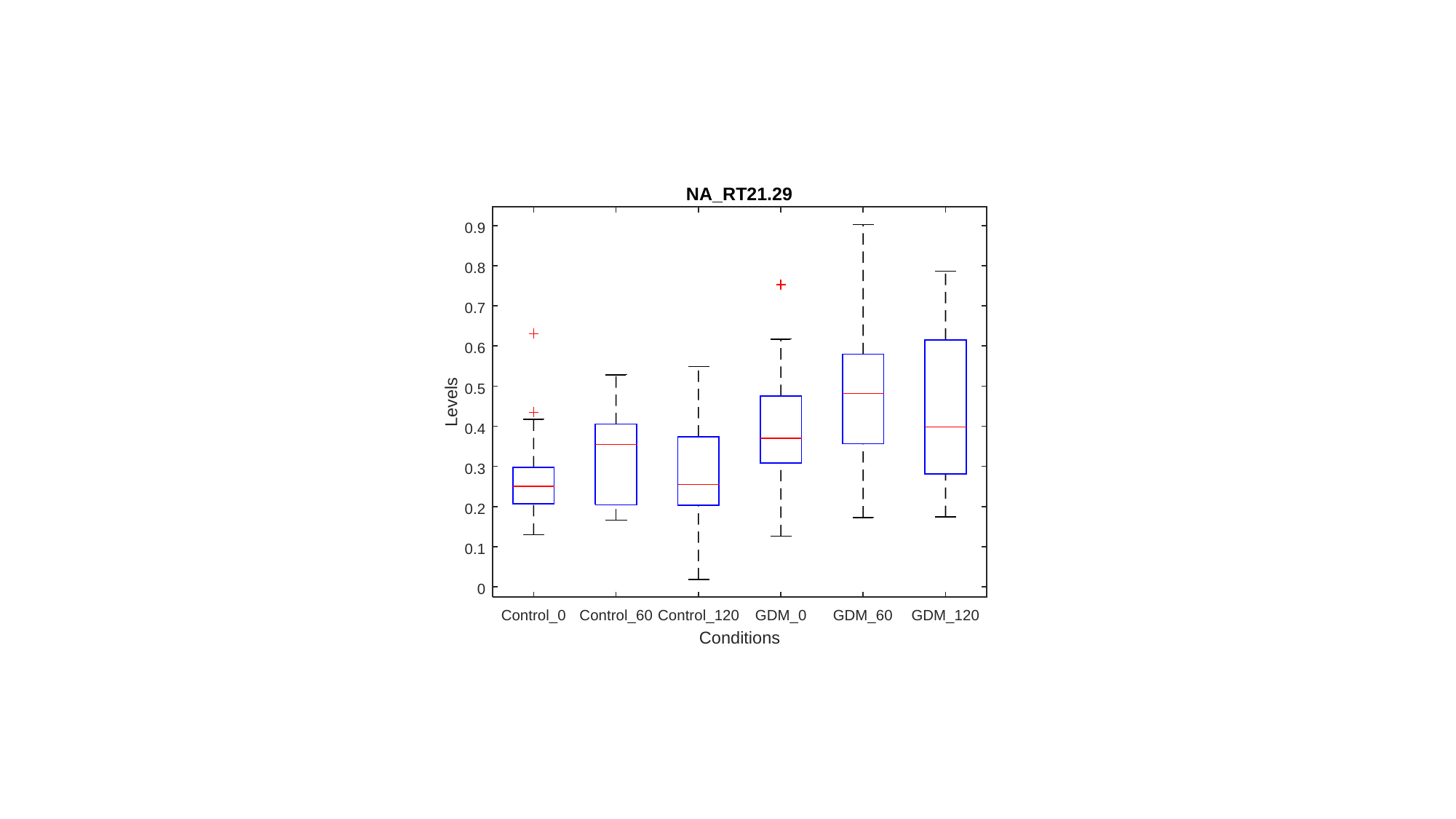

## Slide 20
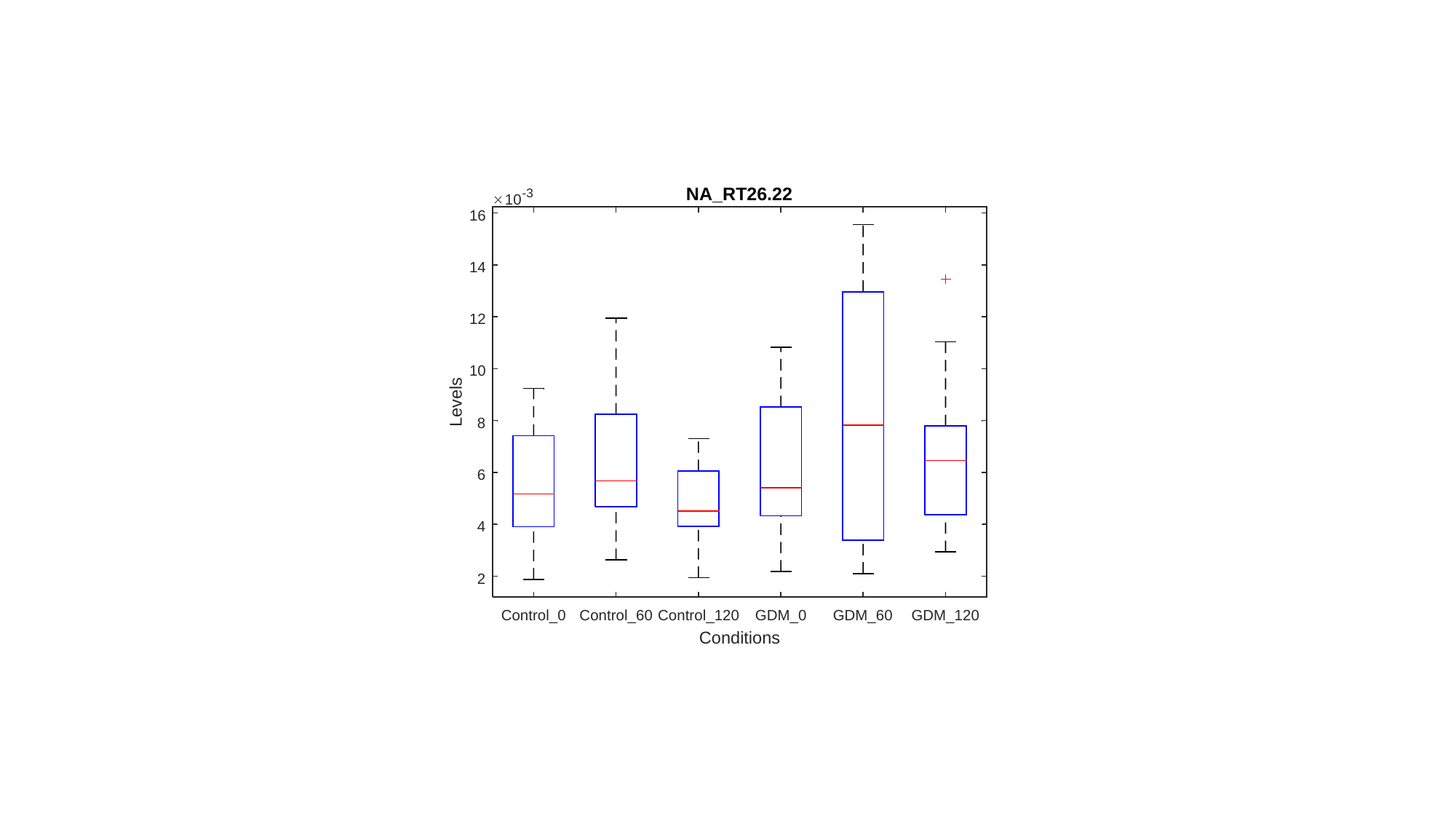

## Slide 21
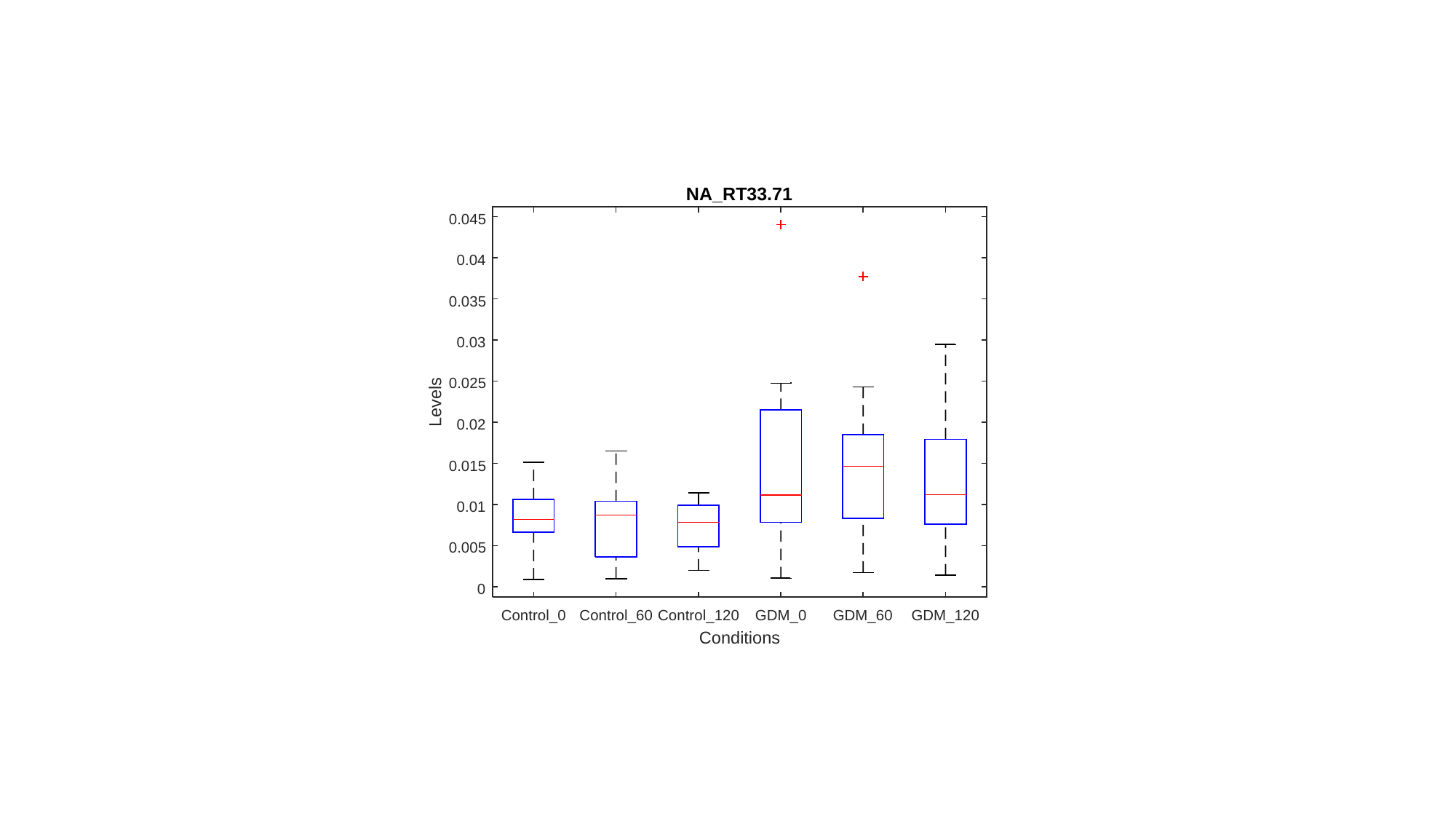

## Slide 22
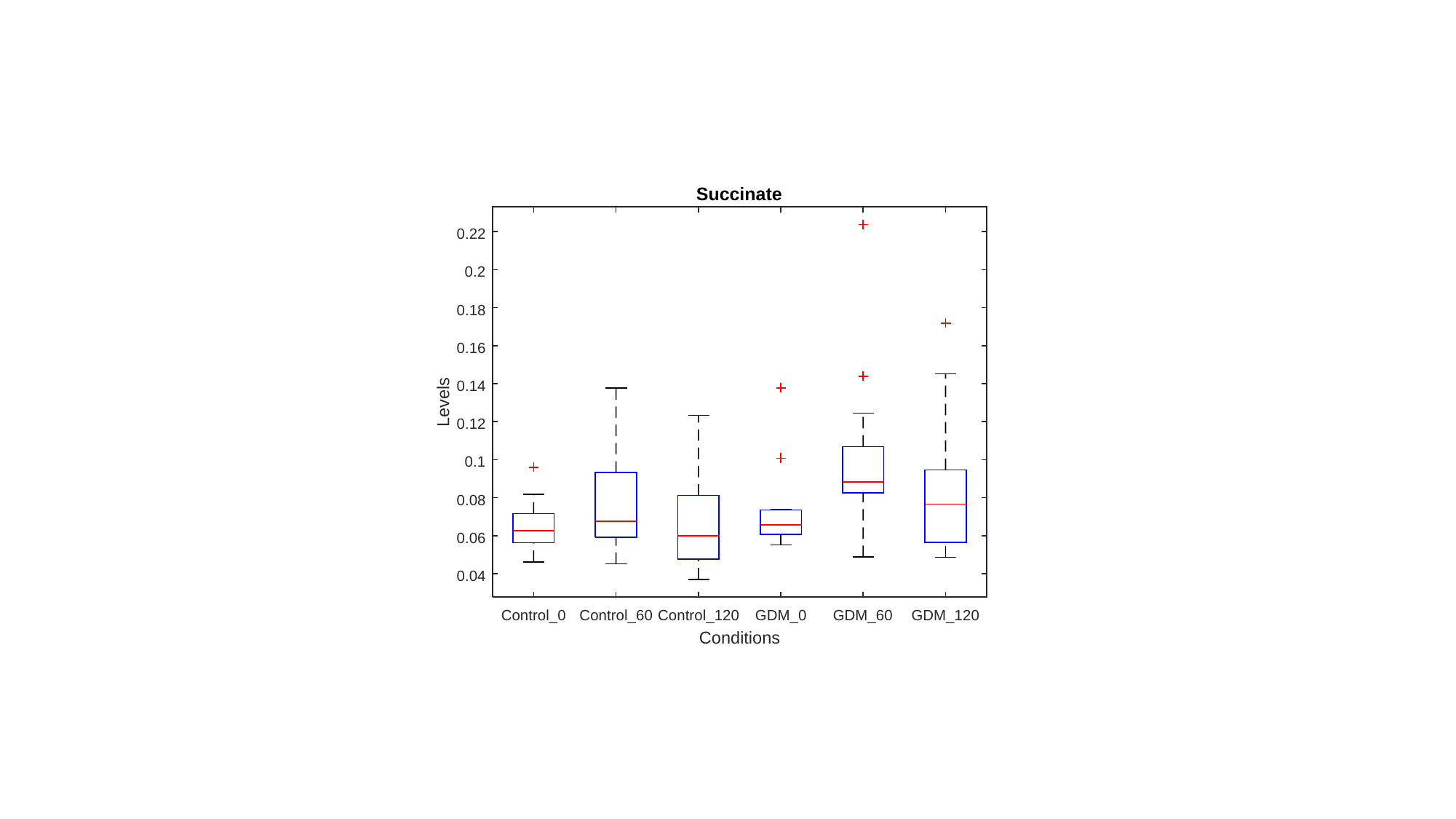

## Slide 23
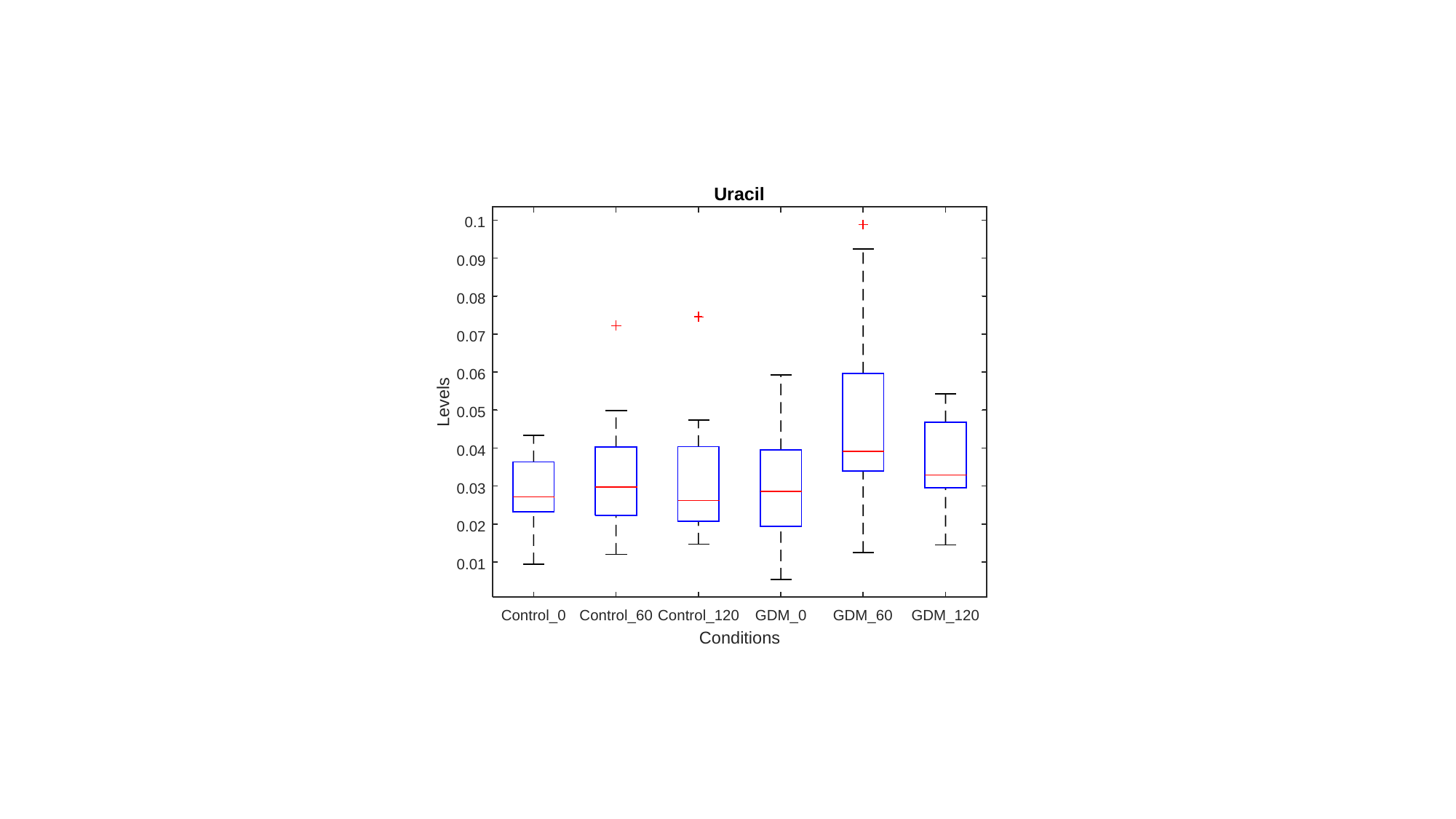

## Slide 24
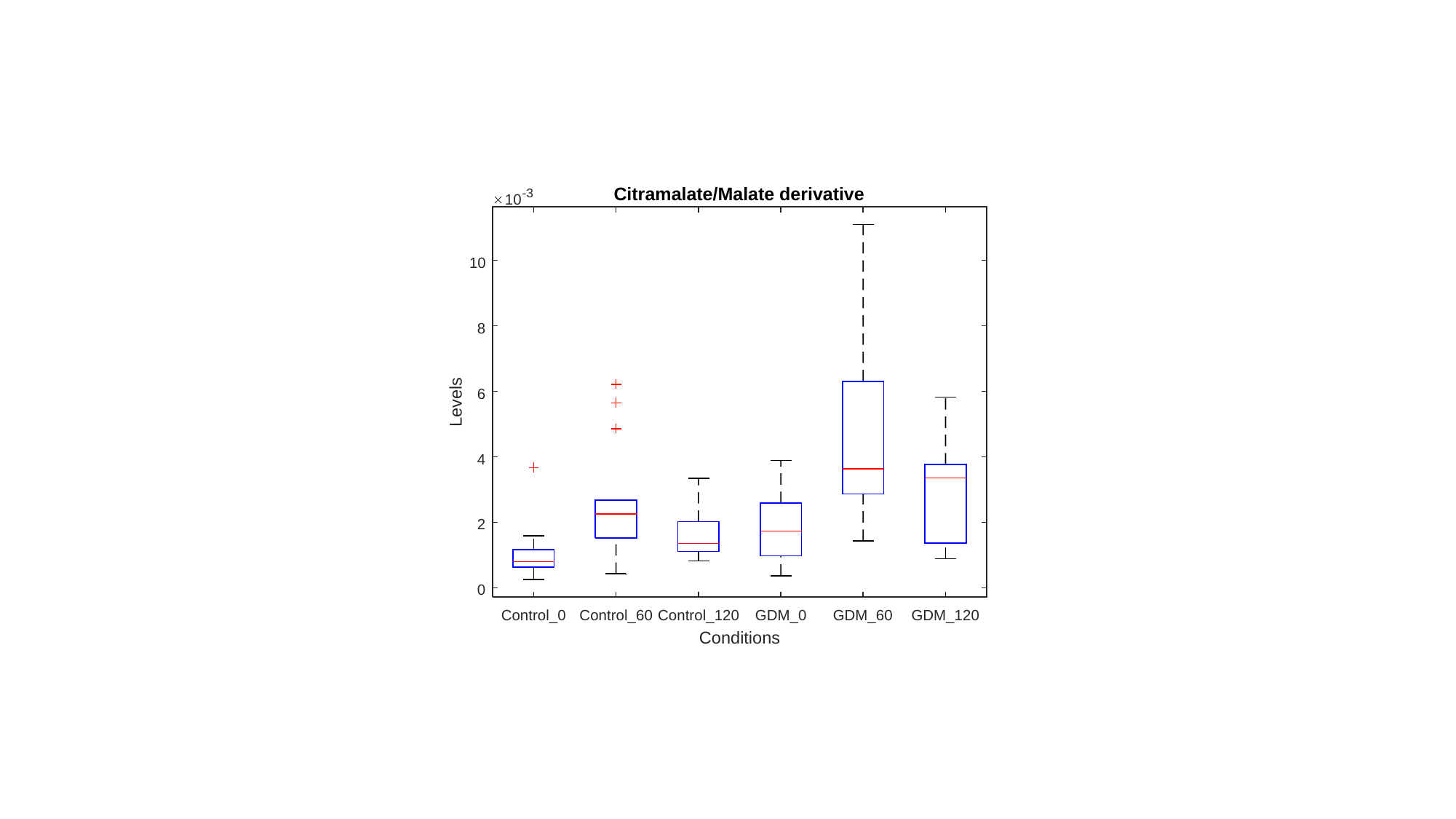

## Slide 25
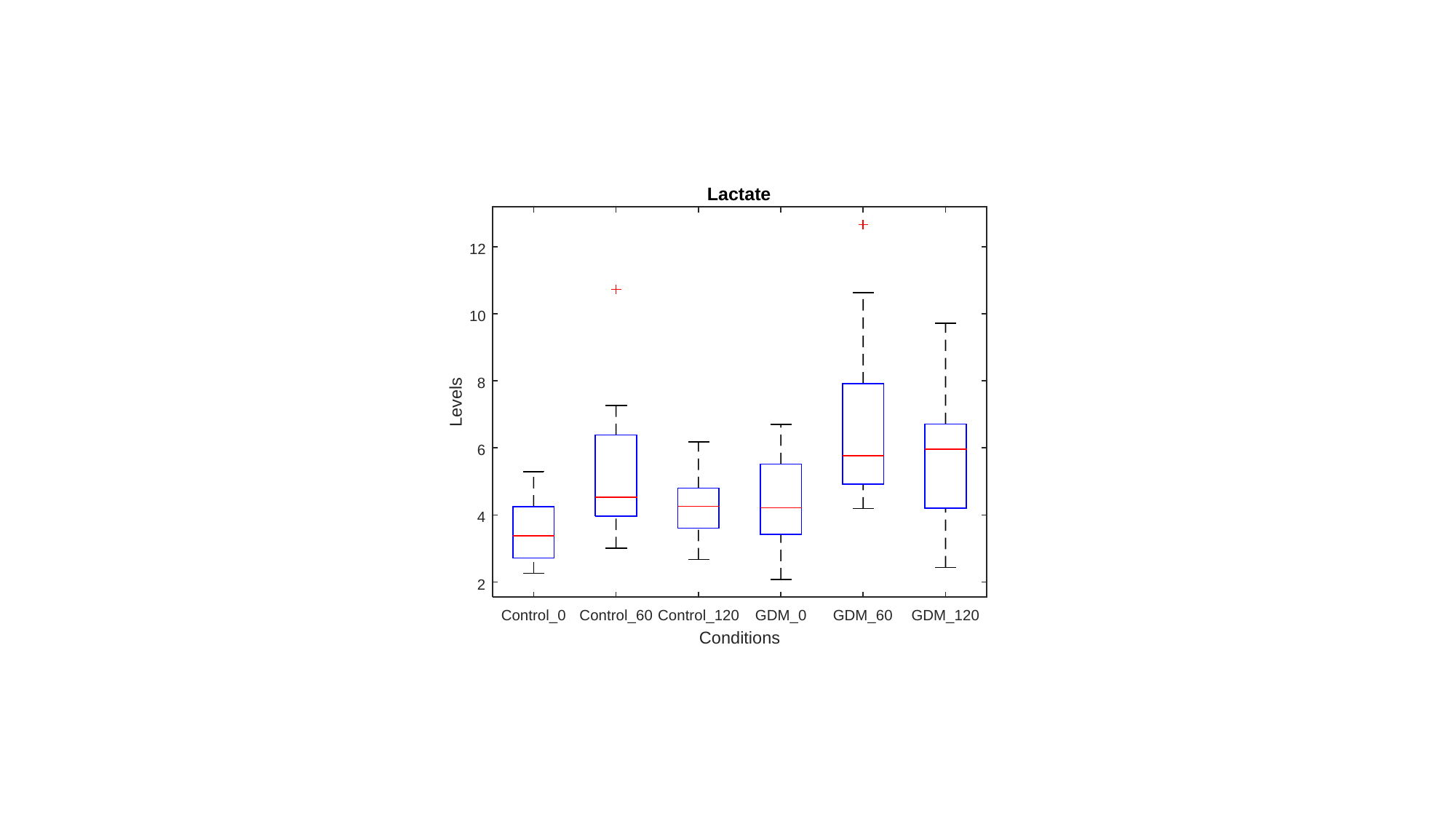

## Slide 26
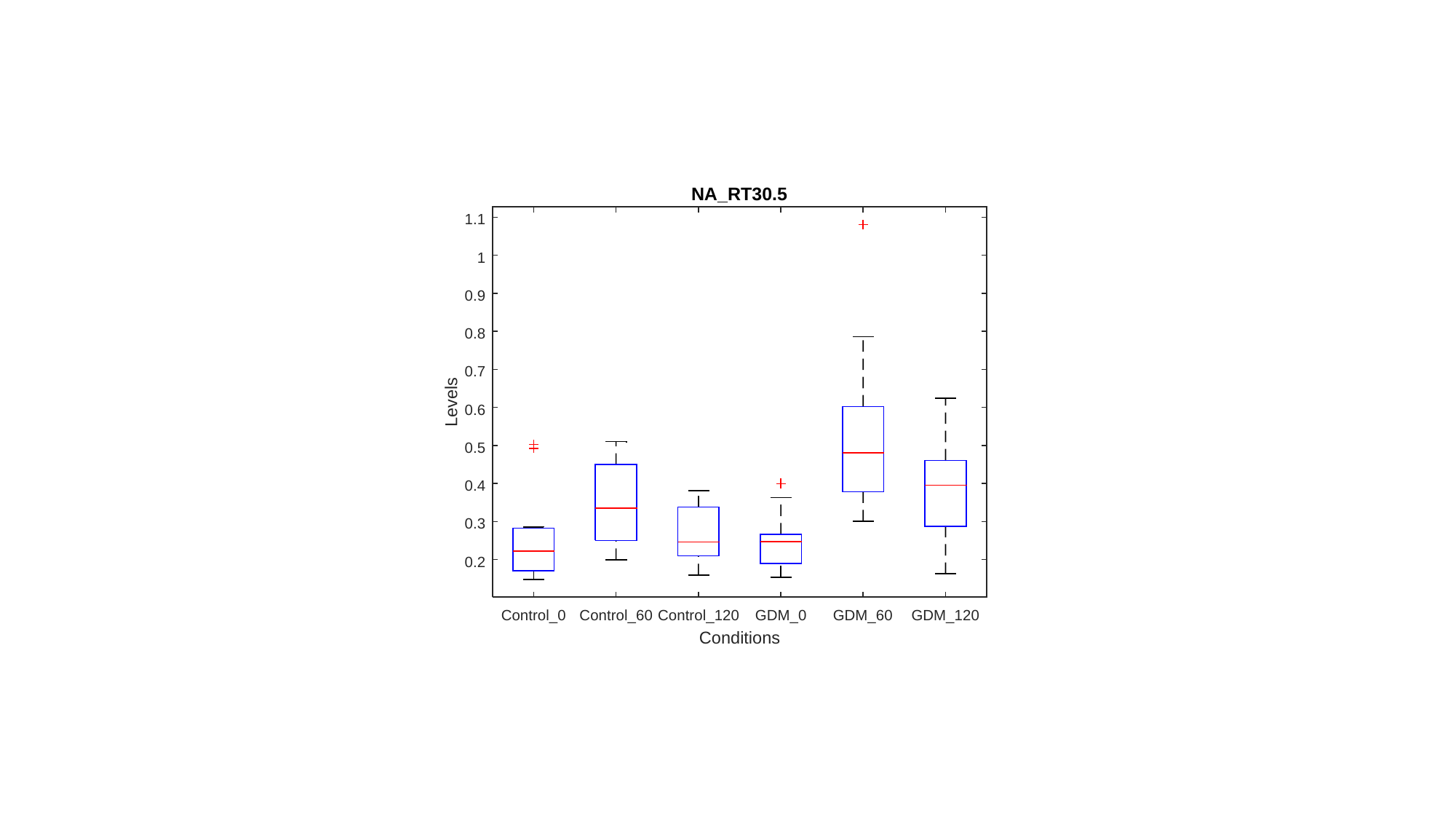

## Slide 27
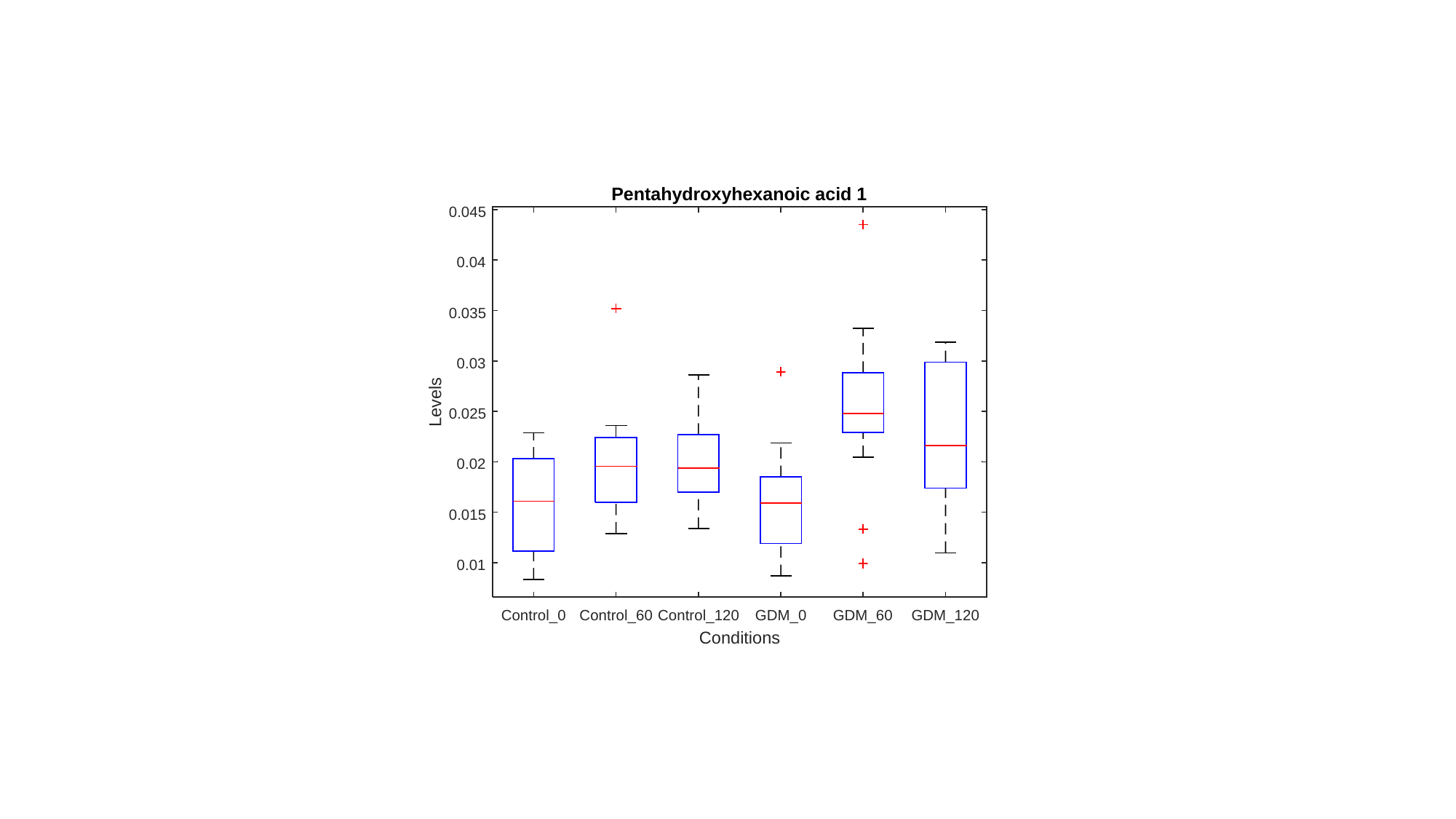

## Slide 28
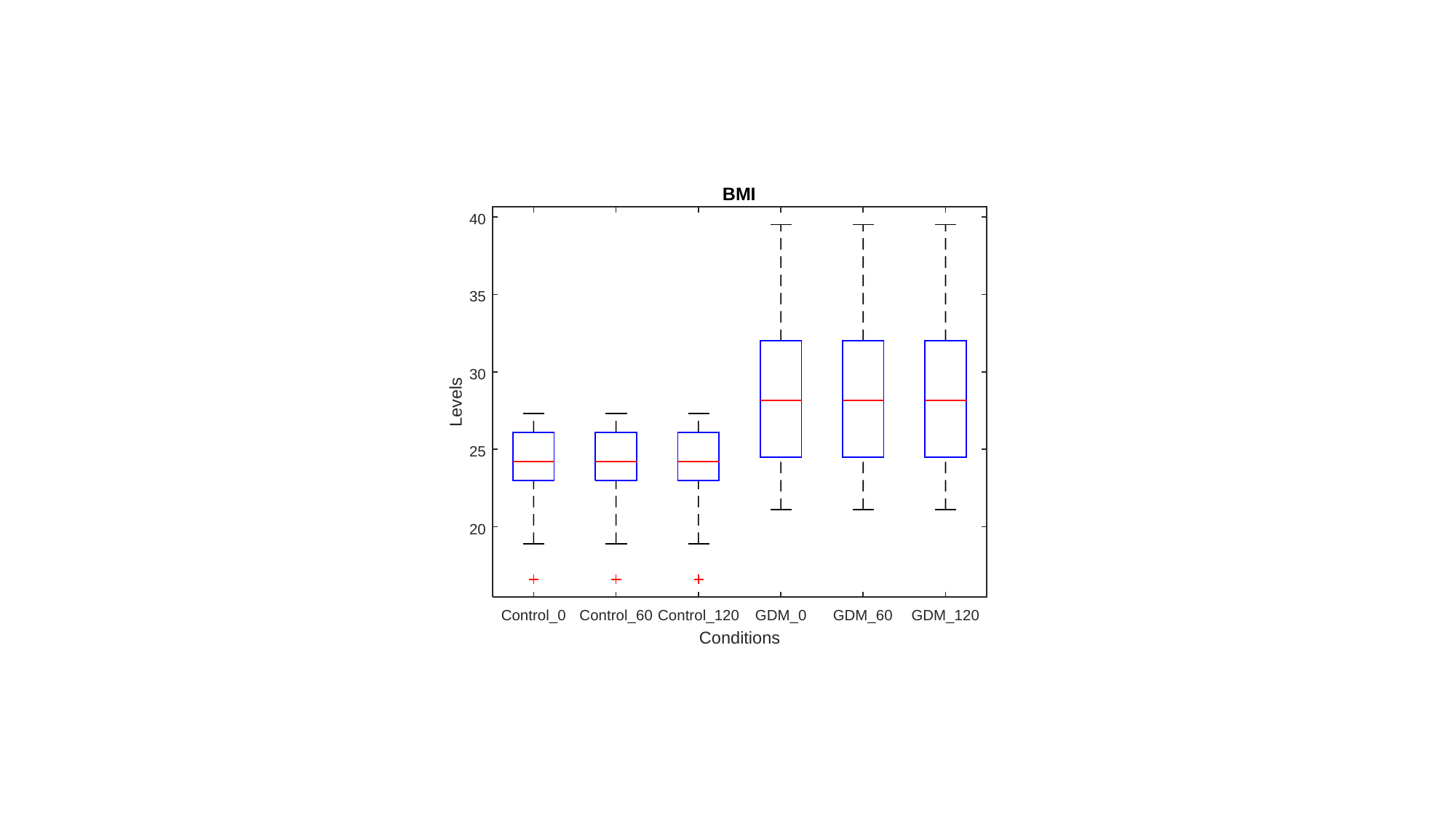

## Slide 29
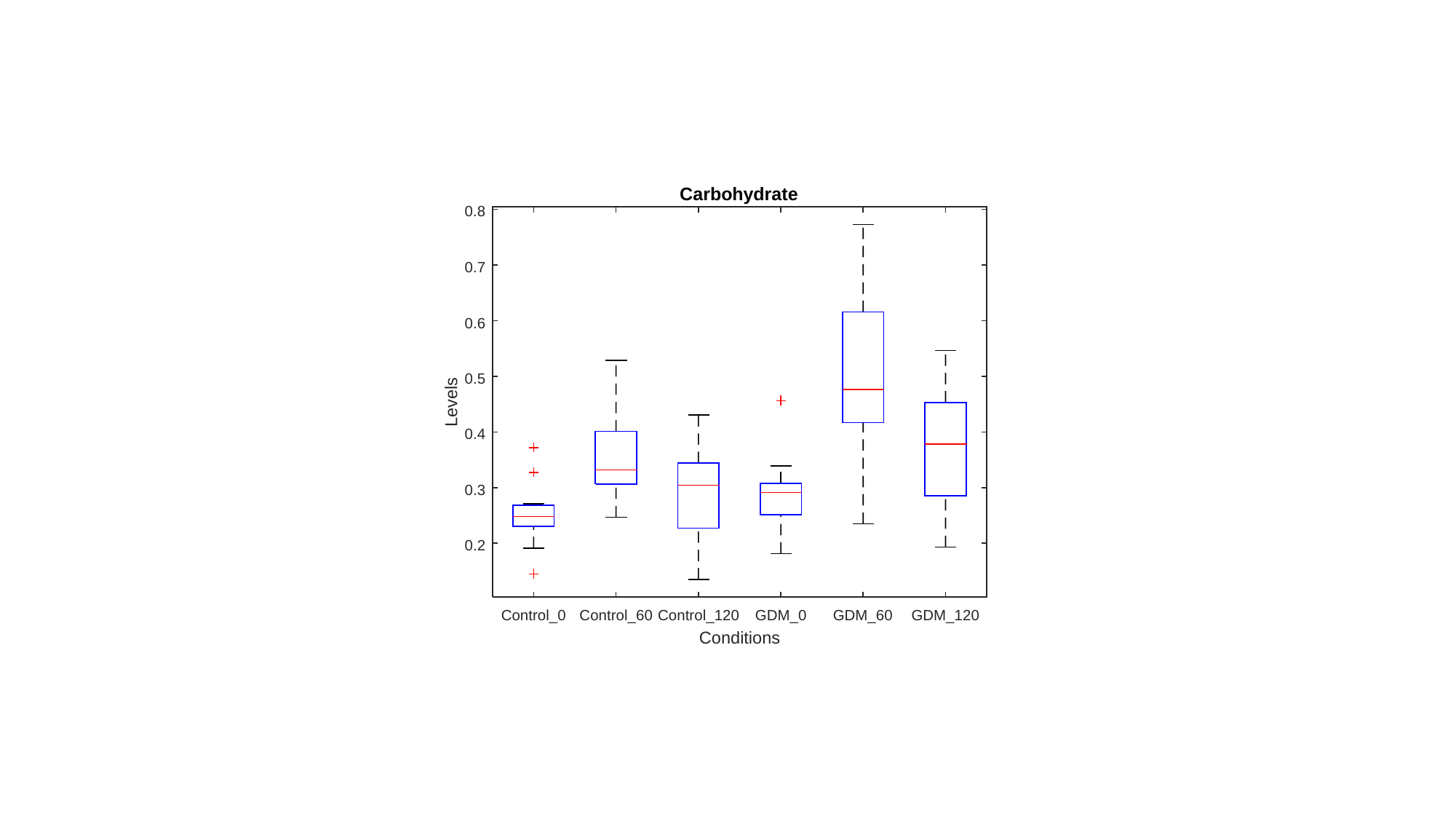

## Slide 30
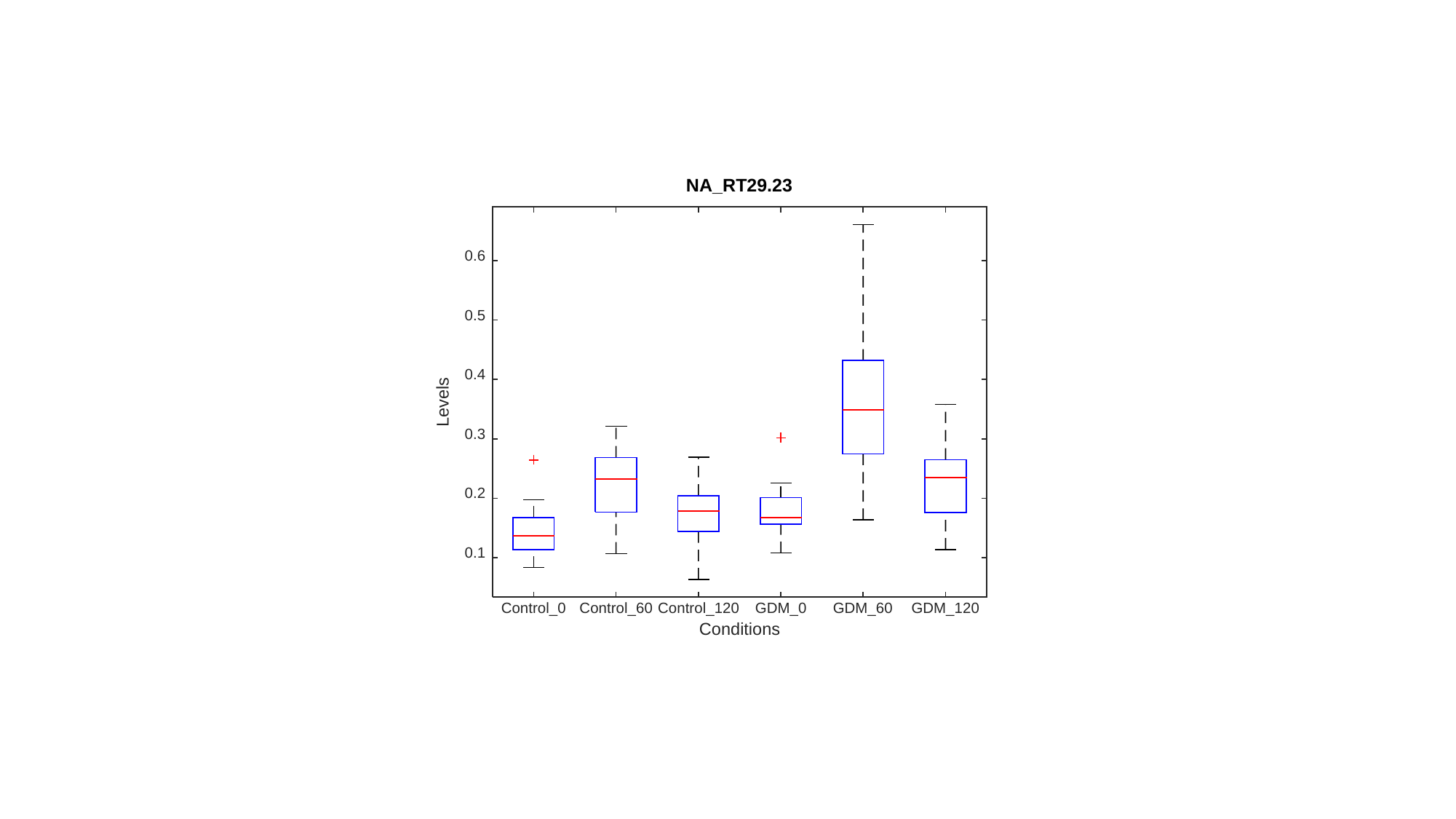

## Slide 31
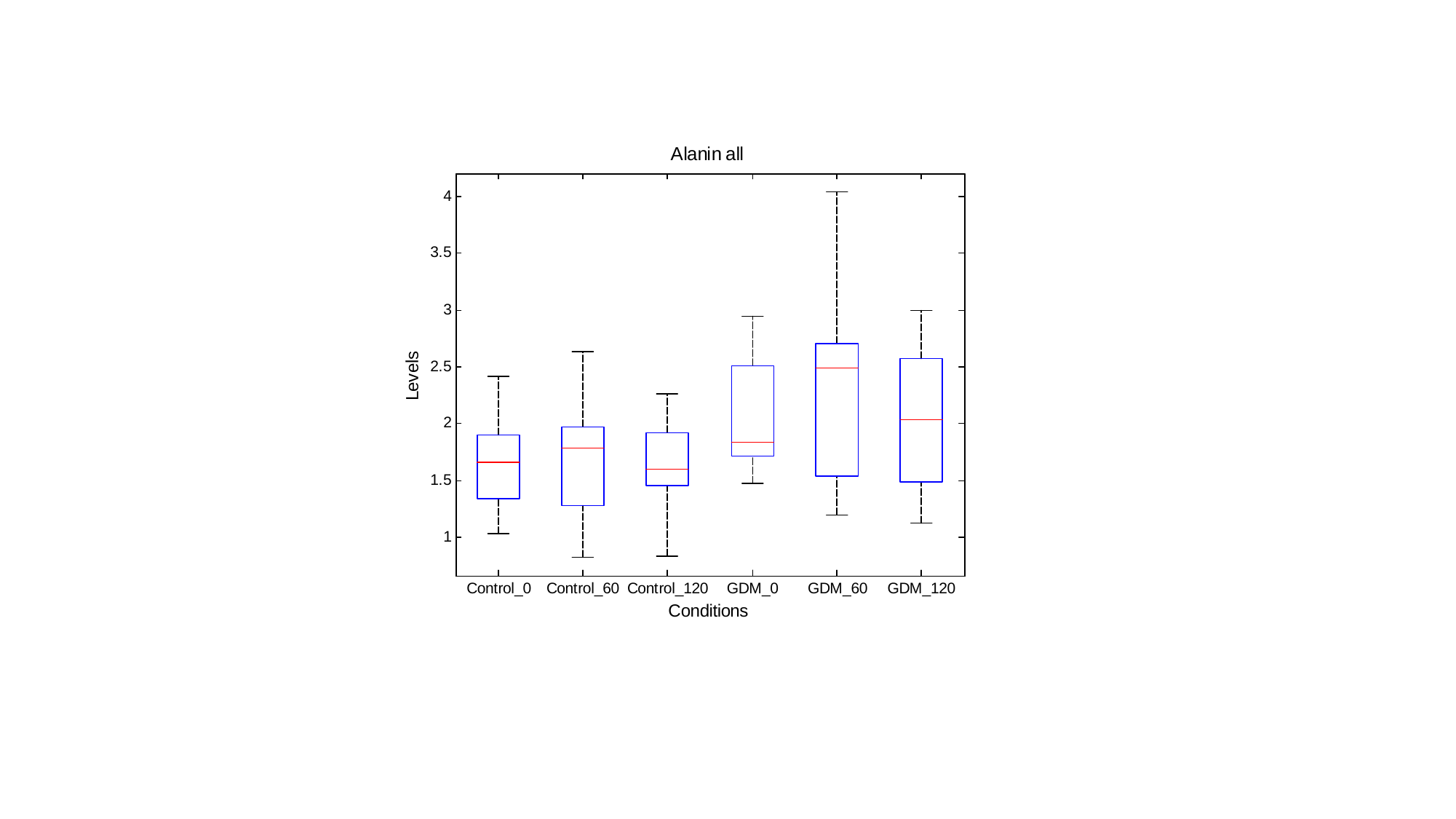

## Slide 32
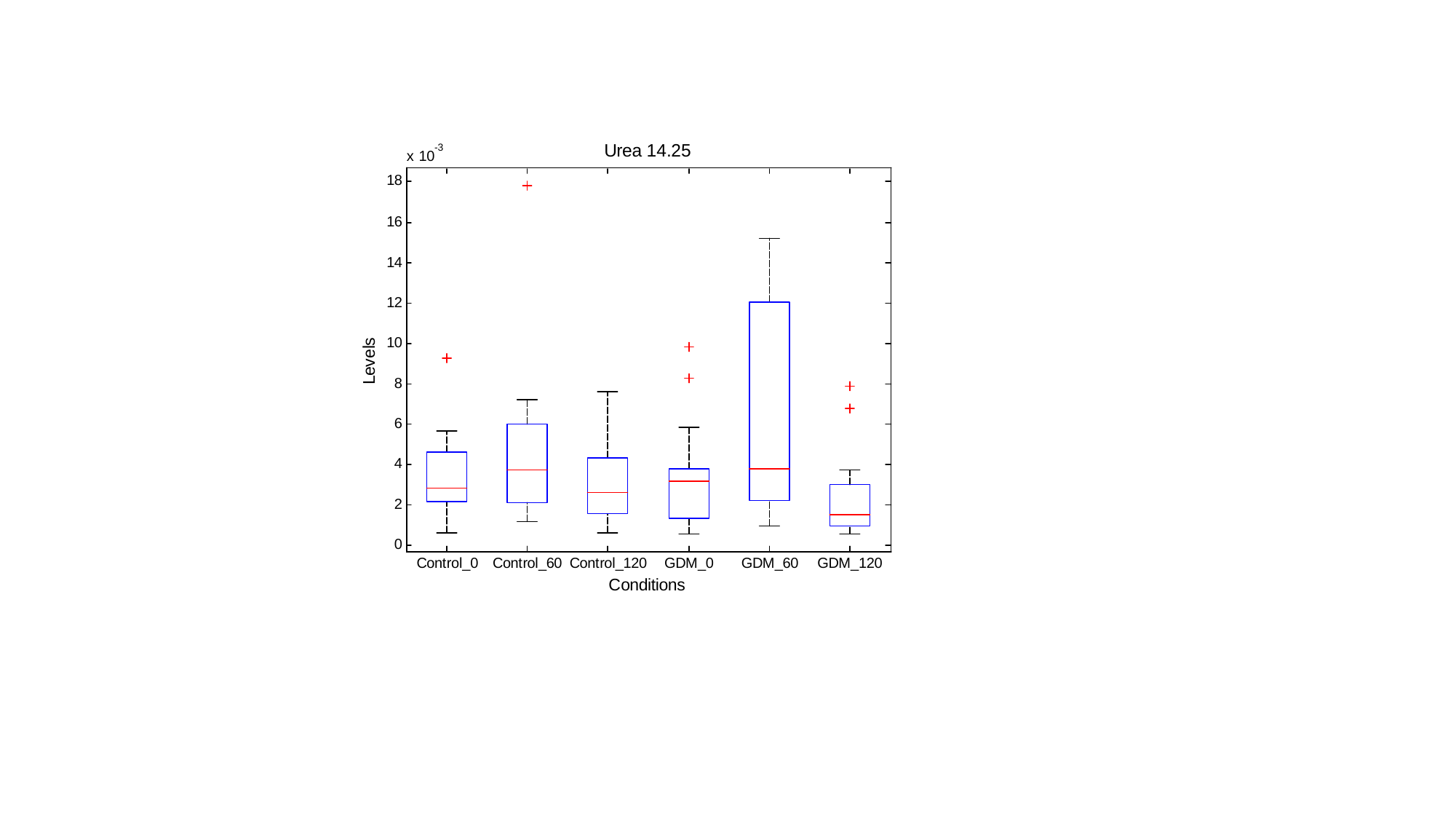

## Slide 33
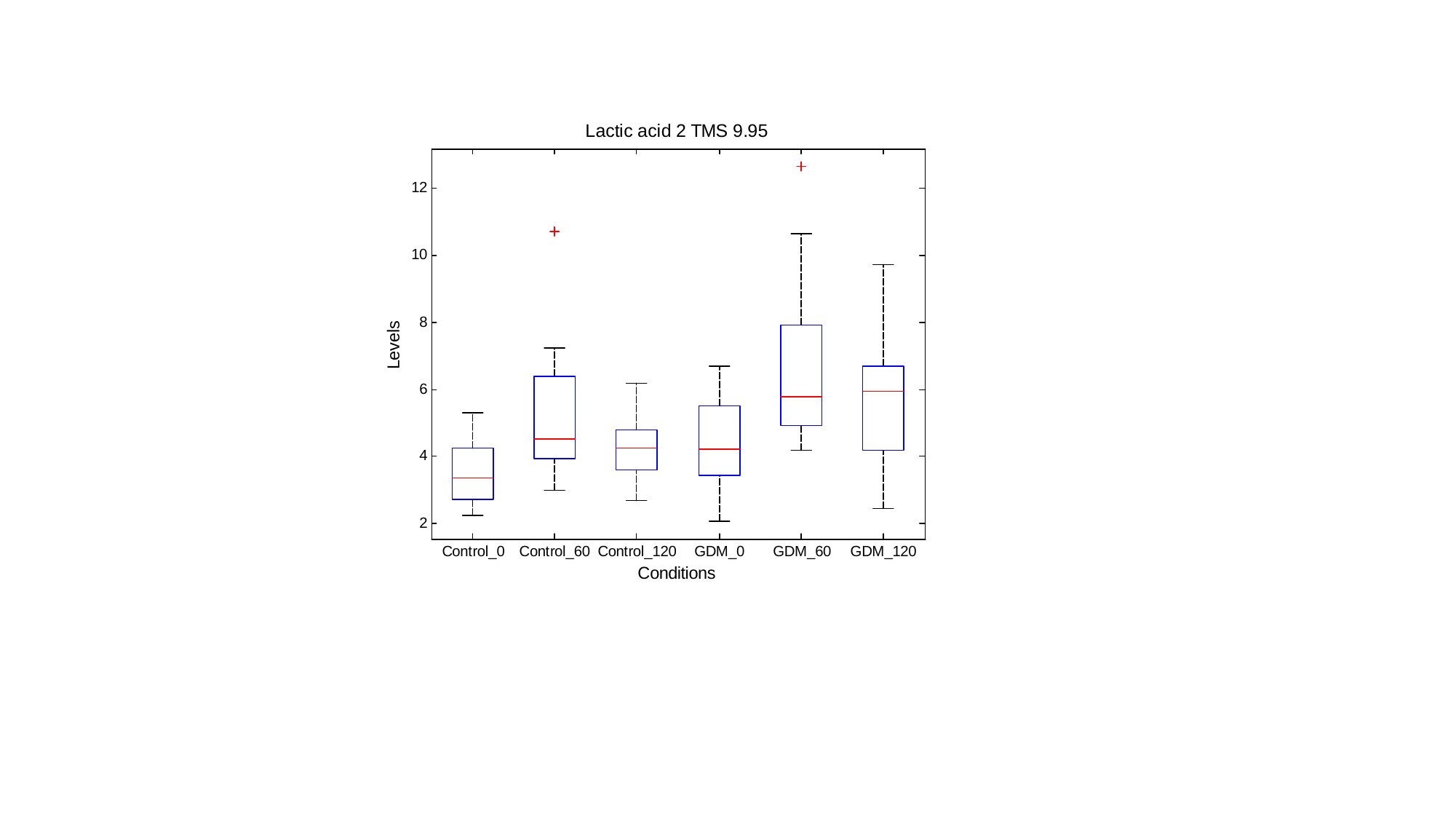

## Slide 34
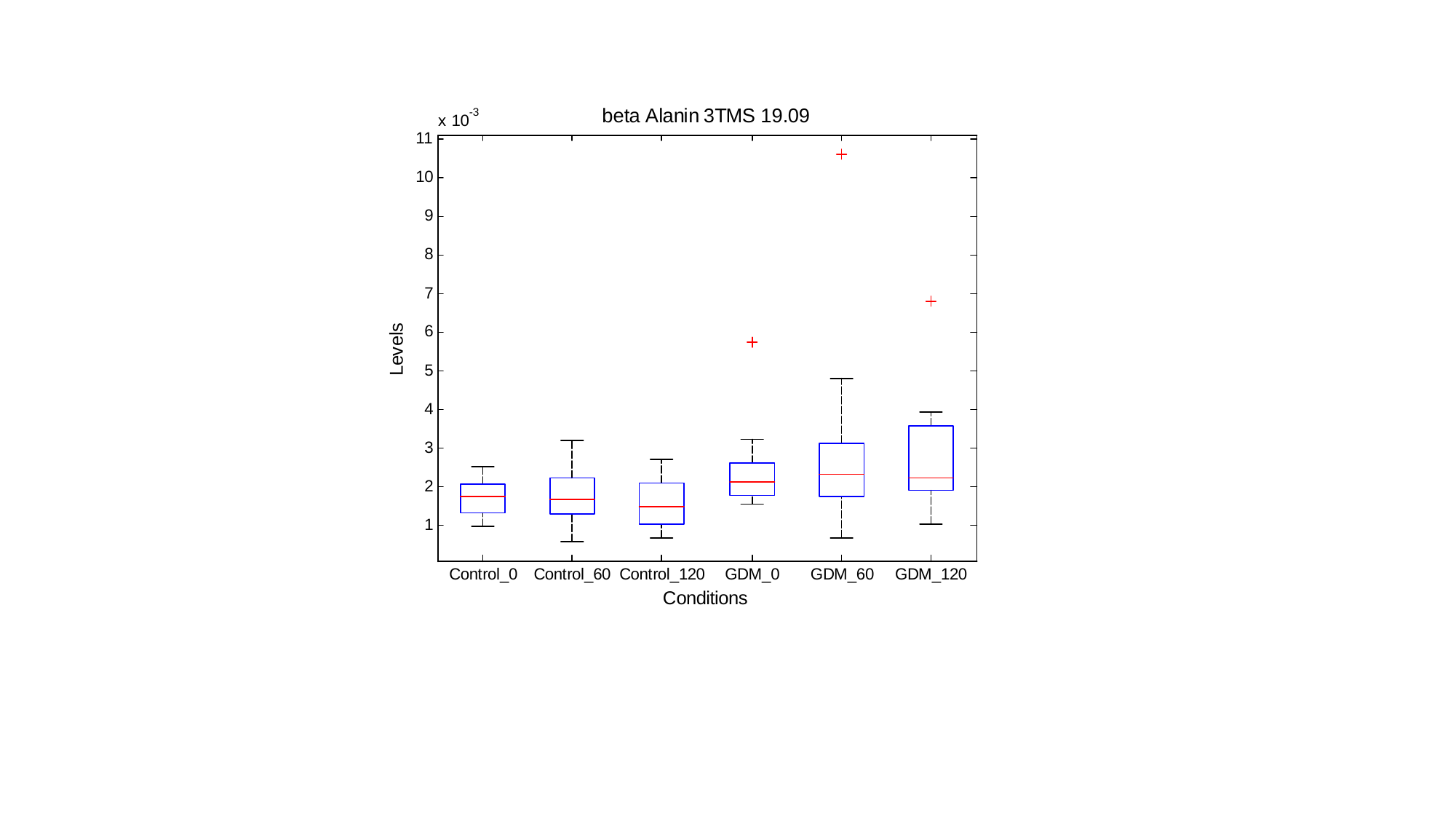

## Slide 35
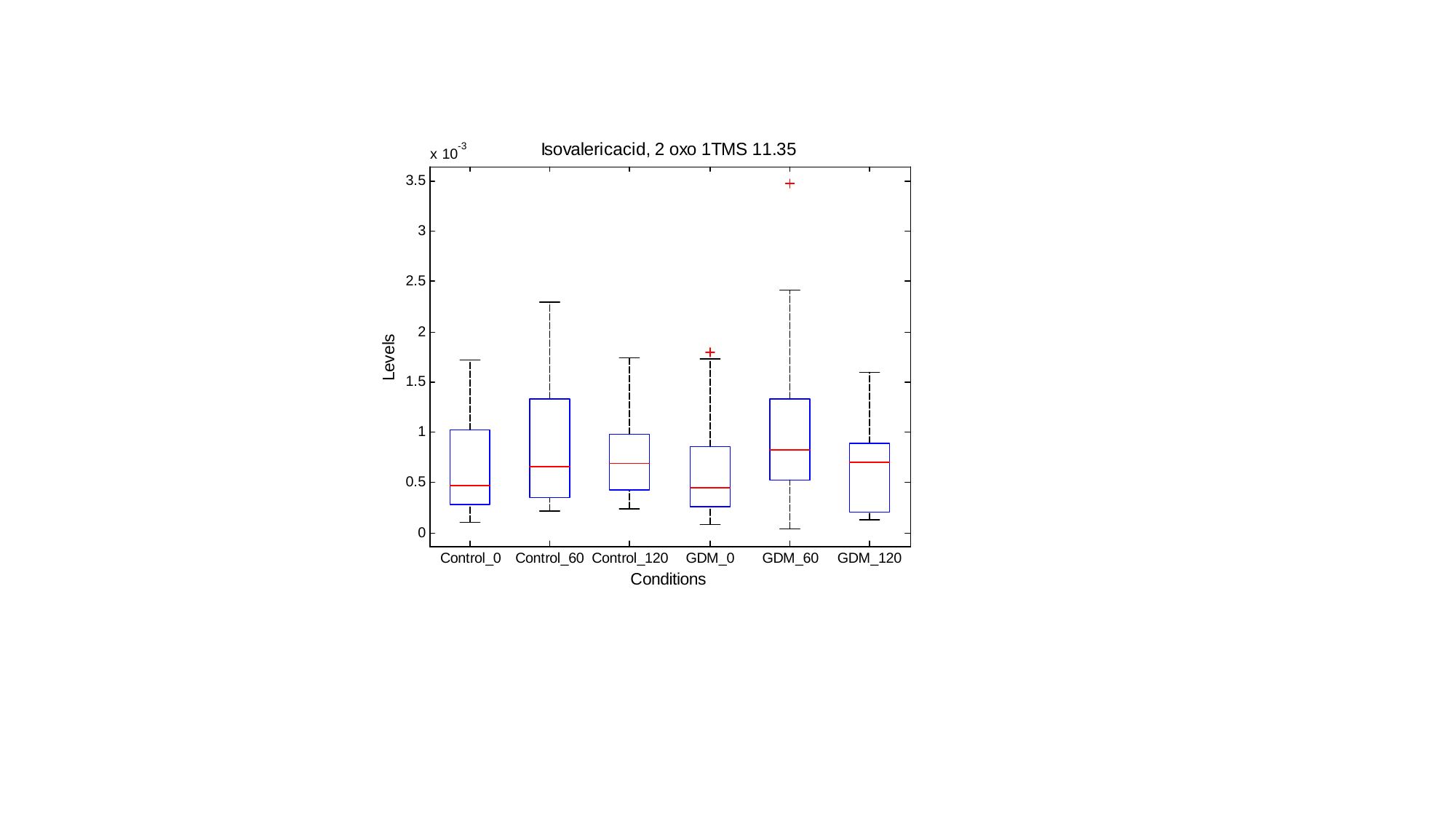

## Slide 36
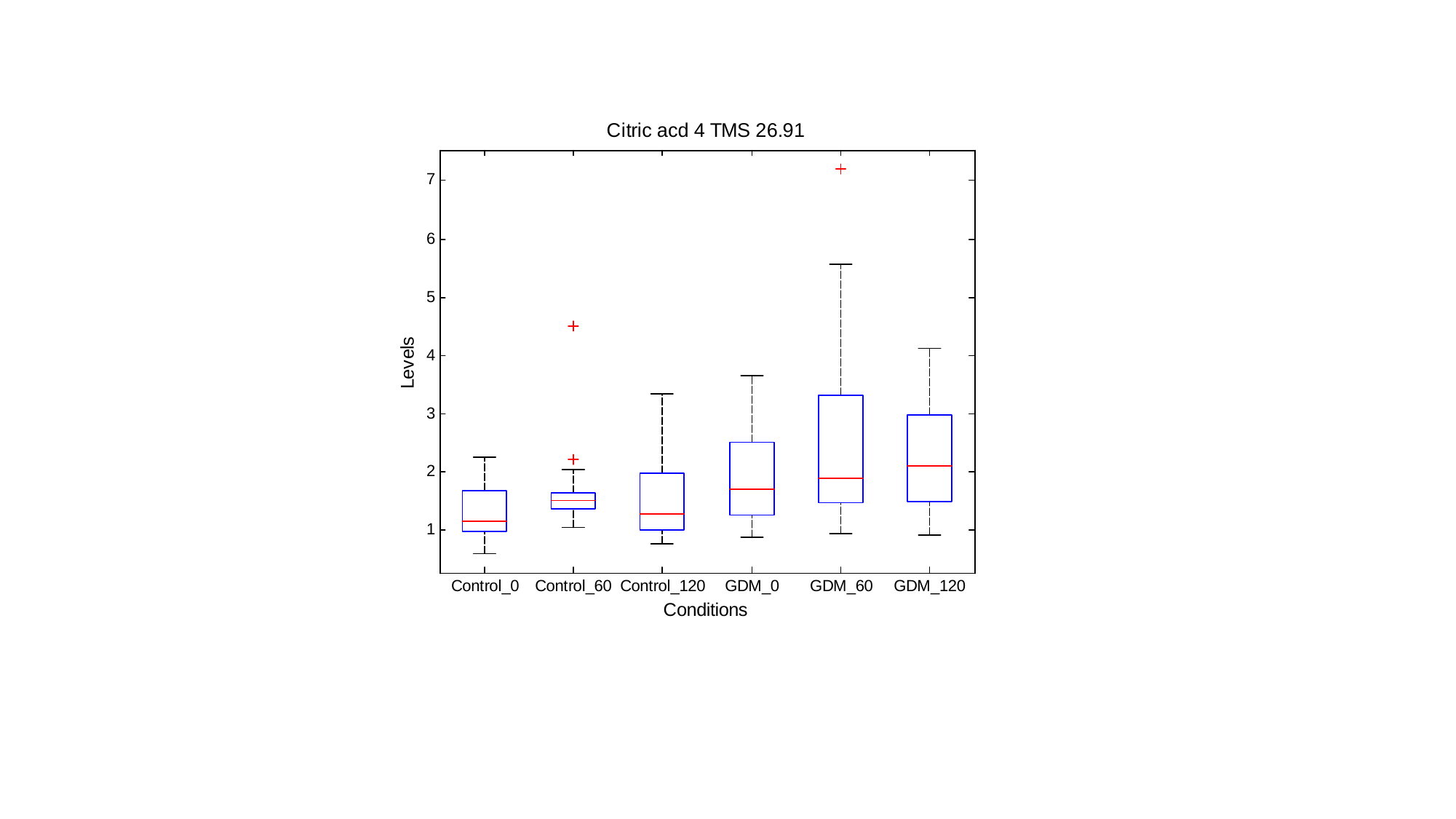

## Slide 37
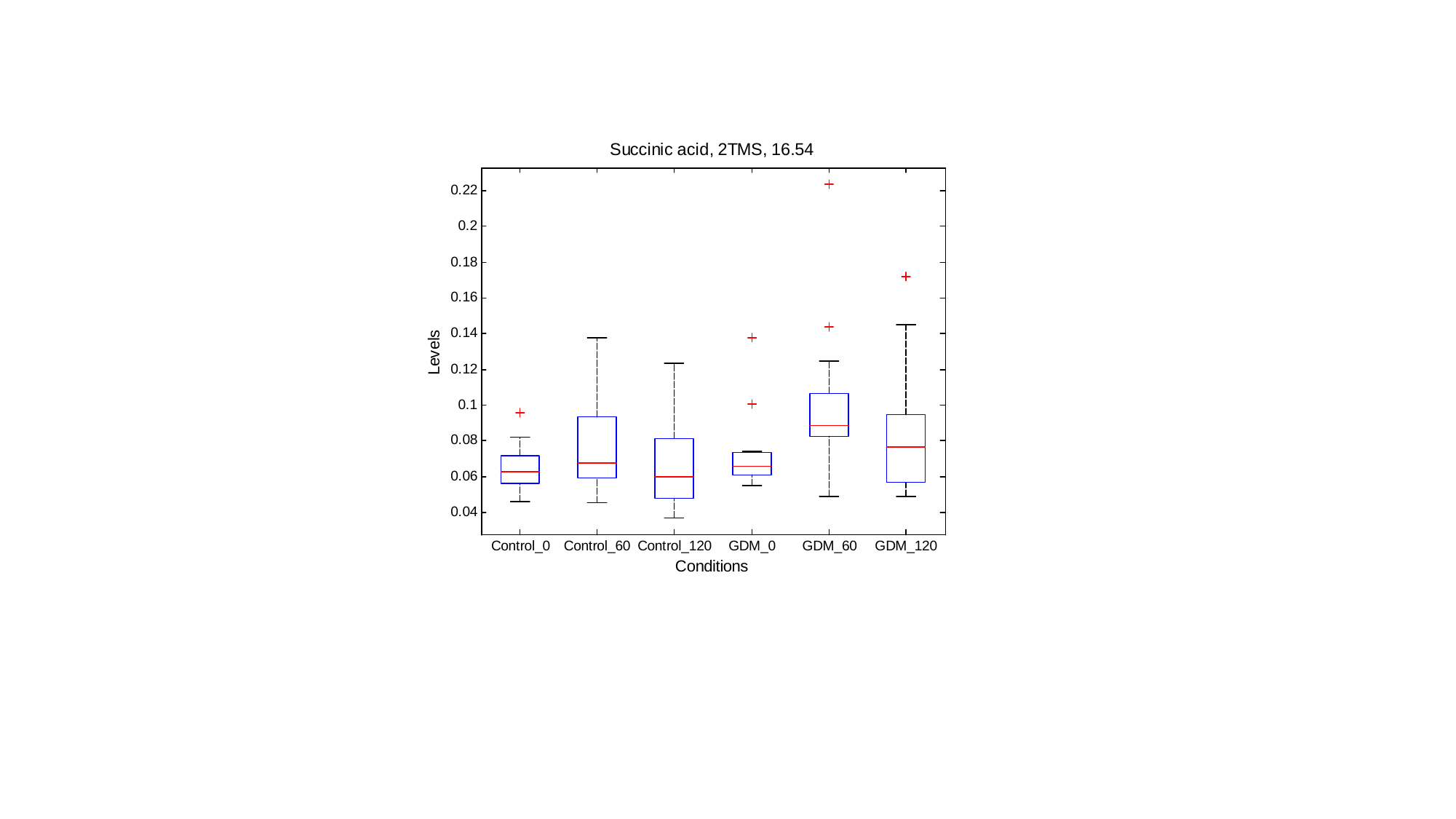

## Slide 38
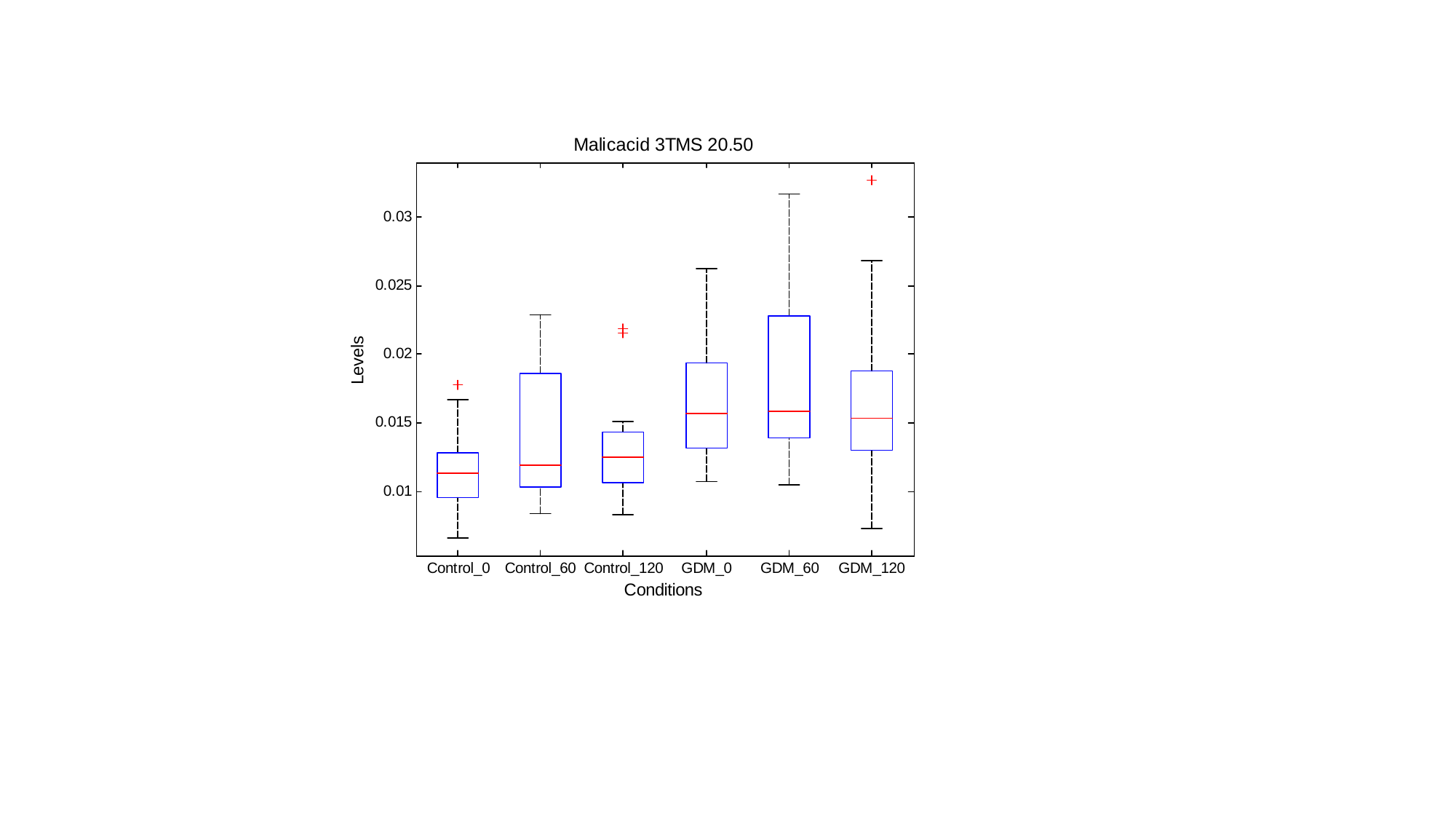

## Slide 39
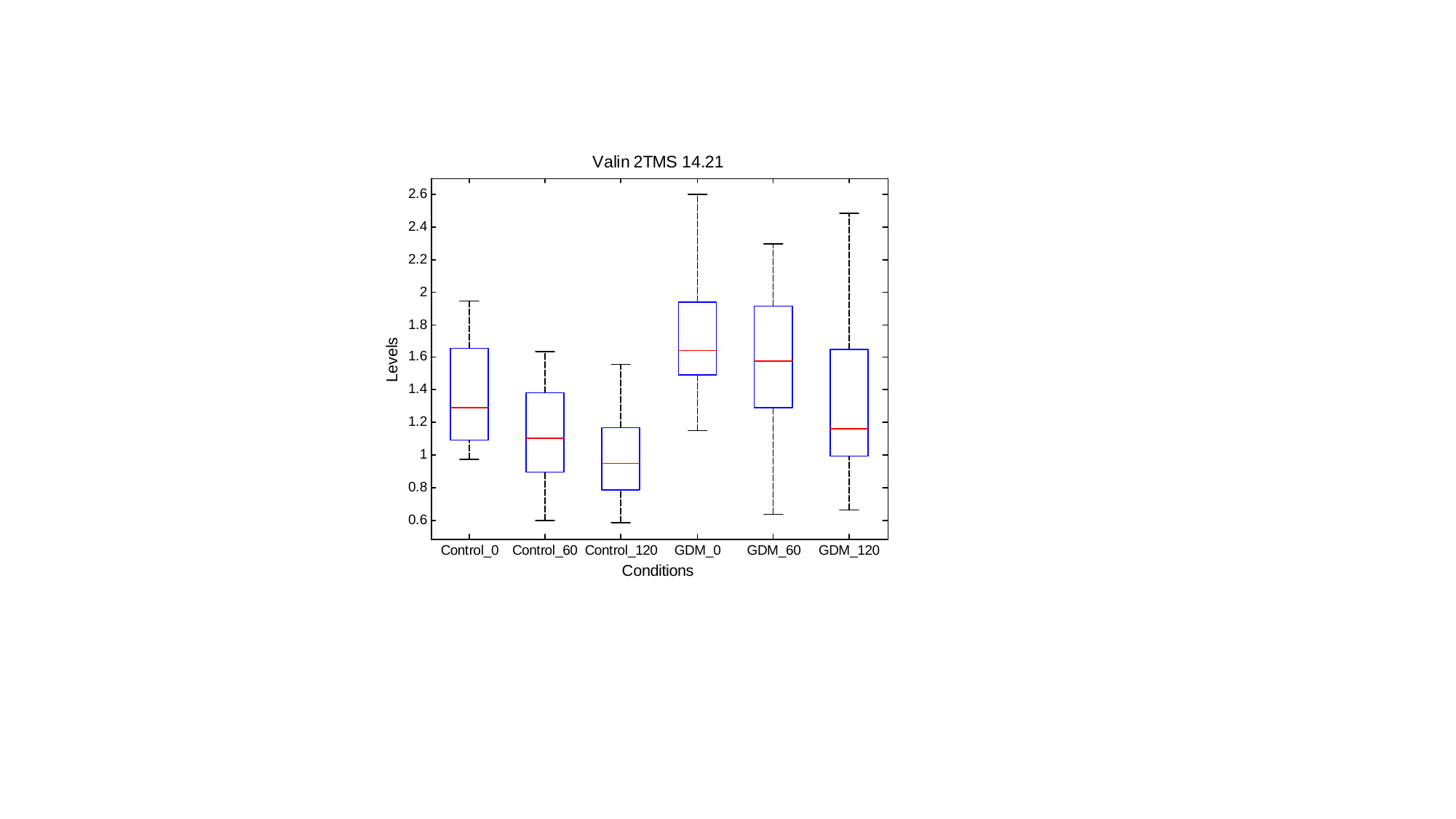

Supplement: Supplementary file 1 [file Presentation1.pptx]
